# RNA-Seq Provides New Insights into the Molecular Events Involved in “Ball-Skin versus Bladder Effect” on Fruit Cracking in Litchi

**DOI:** 10.3390/ijms22010454

**Published:** 2021-01-05

**Authors:** Jun Wang, Xiao Fang Wu, Yong Tang, Jian Guo Li, Ming Lei Zhao

**Affiliations:** 1 State Key Laboratory for Conservation and Utilization of Subtropical Agro-Bioresources, South China Agricultural University, Guangzhou 510642, China; 2015101606@stu.scau.edu.cn (J.W.); 20173088018@stu.scau.edu.cn (X.F.W.); 20173088016@stu.scau.edu.cn (Y.T.); 2 Ministry of Agriculture and Rural Affairs Key Laboratory of South China Horticultural Crop Biology and Germplasm Enhancement, College of Horticulture, South China Agricultural University, Guangzhou 510642, China

**Keywords:** *Litchi chinensis* Sonn., fruit cracking, RNA-seq, WGCNA, ball-skin versus bladder effect

## Abstract

Fruit cracking is a disorder of fruit development in response to internal or external cues, which causes a loss in the economic value of fruit. Therefore, exploring the mechanism underlying fruit cracking is of great significance to increase the economic yield of fruit trees. However, the molecular mechanism underlying fruit cracking is still poorly understood. Litchi, as an important tropical and subtropical fruit crop, contributes significantly to the gross agricultural product in Southeast Asia. One important agricultural concern in the litchi industry is that some famous varieties with high economic value such as ‘Nuomici’ are susceptible to fruit cracking. Here, the cracking-susceptible cultivar ‘Nuomici’ and cracking-resistant cultivar ‘Huaizhi’ were selected, and the samples including pericarp and aril during fruit development and cracking were collected for RNA-Seq analysis. Based on weighted gene co-expression network analysis (WGCNA) and the “ball-skin versus bladder effect” theory (fruit cracking occurs upon the aril expanding pressure exceeds the pericarp strength), it was found that seven co-expression modules genes (1733 candidate genes) were closely associated with fruit cracking in ‘Nuomici’. Importantly, we propose that the low expression level of genes related to plant hormones (Auxin, Gibberellins, Ethylene), transcription factors, calcium transport and signaling, and lipid synthesis might decrease the mechanical strength of pericarp in ‘Nuomici’, while high expression level of genes associated with plant hormones (Auxin and abscisic acid), transcription factors, starch/sucrose metabolism, and sugar/water transport might increase the aril expanding pressure, thereby resulting in fruit cracking in ‘Nuomici’. In conclusion, our results provide comprehensive molecular events involved in the “ball-skin versus bladder effect” on fruit cracking in litchi.

## 1. Introduction

Fruit cracking is a physiological phenomenon where pericarp cracks due to the pressure from fruit internal growth exceeds the strength given by pericarp growth [1]. Fruit cracking is common in fruit crops such as apple [2], citrus [3], peach [4], and grape [5]. The economic value will be lost when fruit undergo cracking. Therefore, exploring the mechanism underlying fruit cracking has always been an important agricultural concern.

The occurrence of fruit cracking is closely related to internal or external environmental factors, of which calcium nutrition, temperature, and humidity are considered as the main physiological factors [6]. In general, calcium deficiency, low temperature, high air humidity, and soil drought will cause fruit cracking. Consistently, calcium spraying can enhance the pericarp strength via increasing the content of calcium in pericarp, thereby reducing the fruit cracking rate in pomegranate, kiwifruit, and litchi [7,8,9]. In pomegranate, if the fruit are exposed to drought stress during the early stage of development, the pulp will accumulate more dry matter and have lower osmotic potential, as a result, the pulp will expand significantly faster than pericarp upon fruit encountering heavy rain during fruit ripening, and fruit cracking occurs [10].

Fruit cracking is also closely associated with genetic factors as early as 1981, when Cuartero et al. pointed out that fruit cracking was regulated by a set of genes [11]. To date, it has been accepted that cell wall remodeling genes including *expansin* (*EXP*), *endo-1,4-β-glucanase* (*EG*), *polygalacturonase* (*PG*), and *xyloglucan endotrans-glycosylase* (*XET*) play an important role in the control of fruit cracking since these genes can regulate the mechanical strength of the cell wall. In apple, the earlier expression of *MdEXPA3* in pericarp by fruit bagging can induce the expansion of pericarp cells earlier, thereby decreasing the susceptibility of fruit cracking [12]. In sweet cherry, the expression level of genes related to cell wall remodeling and cuticular wax biosynthesis is higher in the pericarp of ‘Kordia’ than that in ‘Bing’, the difference between the expression of these genes may partially explain why ‘Kordia’ is more resistant to fruit cracking than ‘Bing’ [13]. Recently, RNA-Seq on both cracking-resistant and cracking-susceptible cultivars identified genes encoding *XET1*, *XET2*, *glycerolipid triacylglycerol* (*TAG*), and *MADS box transcription factors* (*MDP*) involved in fruit cracking in watermelon [14]. In addition, the decrease in the expression of *PG* and *EXP* via the RNA interference (RNAi) approach in tomato can promote generation of cuticular wax, protochitin, and cellulose in pericarp to increase its extensibility, therefore reducing the fruit cracking rate [15]. Taken together, though these findings reveal that genes related to cell wall remodeling, wax biosynthesis, and calcium signaling play an important role in fruit cracking, the molecular mechanism underlying this process is yet largely unknown.

Litchi (*Litchi chinensis* Sonn.), which originated in southern China, is an important tropical and subtropical fruit crop. Now, it is widely cultivated in over 20 countries such as India, Vietnam, Thailand, Madagascar, Australia, and South Africa. The area of industrial litchi cultivation across the world is about 81.9 million hectares, with a total of output of 4.3 million tons. However, fruit cracking has become a limiting factor in the sustainable development of some famous varieties such as ‘Nuomici’, which experiences a fruit cracking rate between 10–30% in general years, but in some special years such as when fruit encounter heavy rain during ripening, the fruit cracking rate is almost 90%. Litchi fruit growth pattern can be clearly divided into two stages. Stage Ⅰ (last for about 50 days post anthesis), which accounts for two thirds of the whole fruit development period, is characterized by a slow growth of pericarp and seed coat, while stage Ⅱ is characterized by a fast growth of aril and embryo [16]. It has been pointed out that the growth of aril begins when the pericarp grows almost to full size as the potential of aril growth is dependent on the space provided by the pericarp. This relationship between the growth patterns of pericarp and aril is called the “ball-skin versus bladder effect” theory [17]. According to this theory, a decrease in pericarp strength will provide a relatively smaller space for aril expansion, fruit cracking will occur when the aril expands, particularly under high temperature or high air humidity, and or a sudden heavy rain since these environmental conditions can facilitate the aril expanding [18]. It has been found that the expression level of two cell wall remodeling genes (*LcXET1* and *LcEG1*) is higher in the pericarp of cracking-susceptible cultivar ‘Nuomici’ than that in the cracking-resistant cultivar ‘Huaizhi’, suggesting that these two genes might be involved in fruit cracking in litchi via regulating the pericarp extensibility [19,20]. Recently, two studies using transcriptome analysis in combination with metabolomic profiling found that the genes related to cell wall, water transport, calcium transport, and plant hormones were differentially expressed during the process of fruit cracking in the cracking-susceptible cultivar ‘Baitangying’ compared with the cracking-resistant cultivar ‘Feizixiao’, and suggested that the changes in the expression of these genes might lead to the decrease in the cell wall stability of pericarp, faster water transport, and the imbalance of endogenous hormones, thereby inducing fruit cracking in ‘Baitangying’ [21,22]. Though these findings provide new clues into the molecular mechanism underlying fruit cracking in litchi, two aspects should be given more attention: (i) the “ball-skin versus bladder effect” exists in litchi fruit cracking, which occurs as a result of interaction between pericarp strength and aril expanding pressure, thus it is necessary to explore the molecular events in both pericarp and aril; and (ii) fruit cracking in litchi, which is not a sudden phenomenon, is closely associated with fruit development at an early stage. Therefore, the differentially expressed genes (DEGs) during early fruit development (prior to fruit cracking) should be coordinated.

Weighted gene co-expression network analysis (WGCNA), which can connect gene expression patterns with phenotypes, has become a powerful strategy to identify key candidate genes that are correlated to specific phenotypic traits [23]. Thus, in this study, we selected the cracking-susceptible litchi cultivar ‘Nuomici’ and cracking-resistant cultivar ‘Huaizhi’ as materials, and samples including both pericarp and aril were collected from developing fruit and fruit that are under cracking for RNA sequencing. We then connected the differentially expressed genes with the daily cracking rate (DCR) by WGCNA to obtain the key modules and genes involved in fruit cracking. Finally, a preliminary model of molecular events in control of fruit cracking in ‘Nuomici’ litchi was discussed.

## 2. Results

### 2.1. Dynamic of Fruit Cracking Rate between ‘Nuomici’ and ‘Huaizhi’

**Figure 1:**
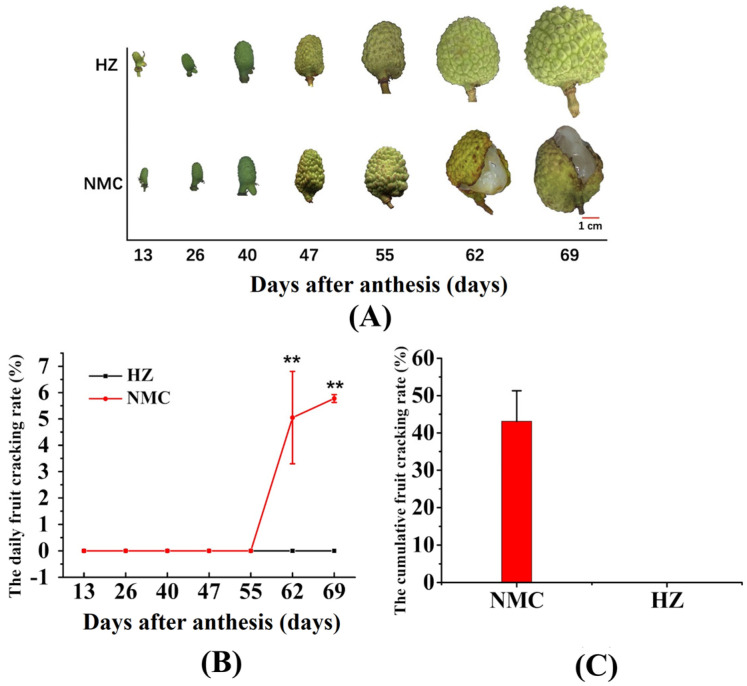
The fruit phenotype at different stages of development (**A**), daily cracking rate (**B**), and the cumulative fruit cracking rate (**C**) between ‘Nuomici (NMC)’ and ‘Huaizhi (HZ)’ litchi. Vertical bars represent the standard error of three biological replicates, significant differences at the 0.01 level are indicated with two asterisks (**) according to the Student *t*-test.

As shown in Figure 1, fruit cracking occurred beginning at 55 days after anthesis in ‘Nuomici (NMC)’ and the daily cracking rate (DCR) was increased thereafter, while the ‘Huaizhi (HZ)’ fruit did not crack throughout the whole fruit growth and development period. The DCR of ‘Nuomici’ was between 55 and 62 days, and 62 and 69 days after anthesis was 5.01% and 5.77%, respectively (Figure 1B). As a result, the cumulative fruit cracking rate of ‘Nuomici’ was as high as 43.12% (Figure 1C).

### 2.2. Transcriptome Sequencing, Mapping, and Annotation

RNA was extracted from pericarp and aril, respectively, and quality was analyzed using an Agilent 2100 bioanalyzer. Subsequently, sixty RNA samples were sequenced with an Illumina HiSeq 2500. After stringent quality assessment and data filtering, 646 million reads were produced by 150 bp paired end sequencing (Table 1). The reads with base quality greater than Q30 (Q ≥ 30) and no ambiguous “N” were defined as high-quality. Approximately 78% of the reads (502 million mapped reads) were successfully aligned to the litchi genome using hisat software (Table 1).

**Figure 2:**
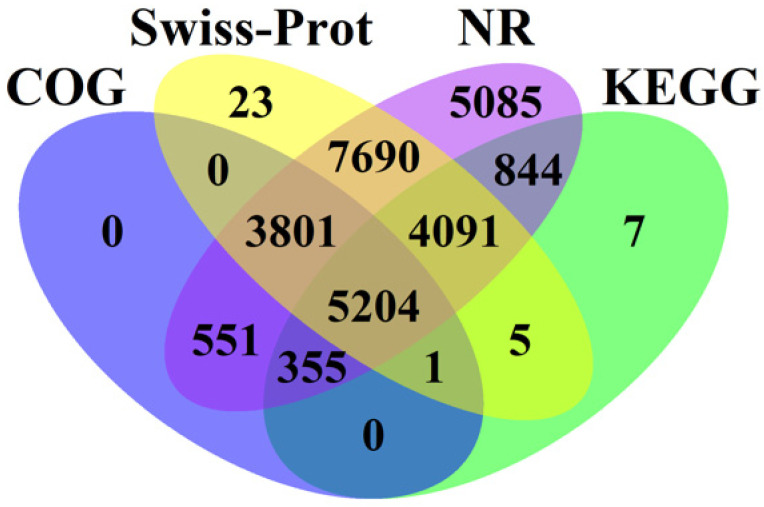
A Venn diagram showing the number of genes annotated by different databases including NR (RefSeq non-redundant proteins), SWiss-Prot (Swiss-Prot Protein Sequence Database), COG (cluster of orthologous group), and KEGG (Kyoto Encyclopedia of Genes and Genomes).

For annotation, the gene expressed in pericarp and aril were compared against four databases (NR: RefSeq non-redundant proteins, SWiss-Prot: Swiss-Prot Protein Sequence Database, COG: cluster of orthologous group, and KEGG: Kyoto Encyclopedia of Genes and Genomes) using BLASTX. A large number of litchi genes including 27,621 genes in Nr, 20,815 genes in Swiss-Prot, 10,507 genes in KEGG, and 9912 in COG were annotated (Figure 2), of which 5204 genes could be shared by these four databases, and most genes could be annotated at whatever two databases.

### 2.3. Screening of Genes Related to Fruit Cracking by Weighted Gene Co-Expression Network Analysis (WGCNA)

**Figure 3:**
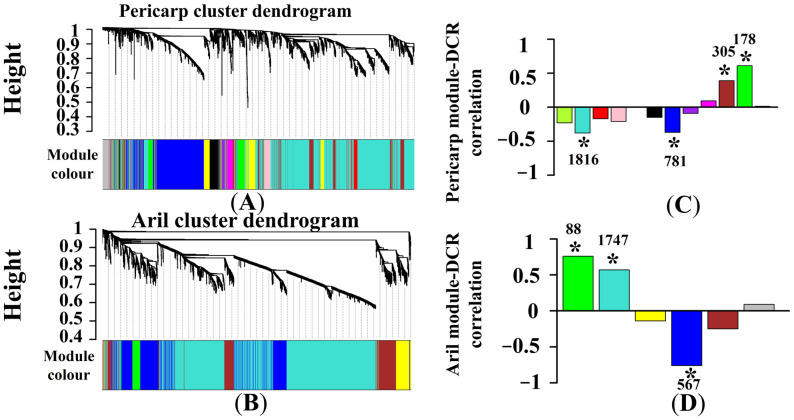
Network construction and hierarchical clustering of differentially expressed genes (DEGs) in pericarp and aril. Genes with similar expression patterns were clustered. Each branch in the (**A**) and (**B**) represents one gene, and the color below each branch represents the co-expression module. Twelve and six modules in (**C**) and (**D**) are grouped by genes with the same color. The asterisk (*) indicates that the module has a significant correlation coefficient (*p* < 0.05) with DCR. The number above (or below) the asterisk represents the gene numbers in this module.

First, differentially expressed genes (DEGs) in pericarp or aril were screened at the same time point using FPKM (Fragments Per Kilobase Million) values according to differential expression greater than two-fold (False discovery rate, FDR < 0.01, log_2_ > 1 or < −1). Then, the DEGs in pericarp and aril at all time points were integrated and defined as P1 and A1, respectively. Results showed a total of 5624 genes in P1 and 4399 genes in A1. Then, DEGs in P1 and A1 were divided into 12 and six independent modules according to WGCNA, respectively (Figure 3A,B). In pericarp, two module genes (turquoise and blue modules) were found to be significantly negative correlated to DCR, while two module genes (brown and green modules) were significantly positively correlated to DCR. In aril, two module genes (green and turquoise modules) were found to be significantly positively correlated to DCR, and one module (blue module) was significantly negatively correlated to DCR (Figure 3C,D). Here, the DEGs that were significantly correlated to DCR in pericarp (3080 genes) and aril (2402 genes) were defined as PM and AM, respectively.

**Figure 4:**
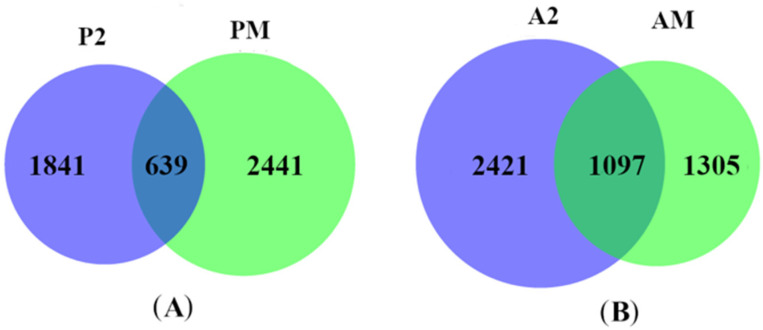
Identification of DEGs during fruit cracking in pericarp and aril. The DEGs in the blue part has a significant correlation coefficient (*p* < 0.05) with DCR, and the DEGs in the green part are genes that showed differential expression in pericarp (**A**) and aril (**B**) between ‘Nuomici’ and ‘Huaizhi’ during fruit cracking.

To further screen DEGs that are more closely related to fruit cracking, we first compared the FPKM values between ‘Nuomici’ and ‘Huaizhi’ in pericarp and aril at 62 d and 69 d after anthesis since the fruit of ‘Nuomici’ were undergoing cracking during this period. We named the DEGs in pericarp (2480 genes) and aril (3158 genes) at these two time points as P2 and A2, respectively. We then compared the DEGs between P2 and PM, A2 and AM. We found that 639 DEGs were shared between P2 and PM and 1097 DEGs were common between A2 and AM (Figure 4). Thus, these DEGs were used for further analysis.

### 2.4. Clustering of Differentially Expressed Genes

**Figure 5:**
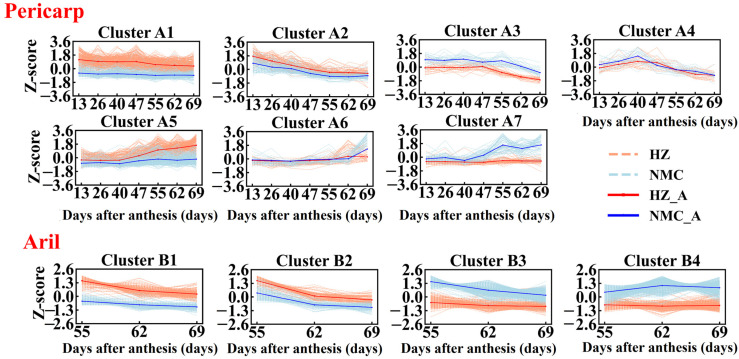
SOTA (Self-Organizing Tree Algorithm) clustering of gene expression patterns. Cluster analysis of DEGs in ‘Huaizhi’ and ‘Nuomici’ based on the Z-score normalized method. The light red dotted and light blue dotted lines represent the expression profiles of ‘Huaizhi’ and ‘Nuomici’ in each cluster, respectively. The red and blue solid line represent the average profile of ‘Huaizhi’ and ‘Nuomici’ in each cluster, respectively.

According to the “ball-skin versus bladder effect” theory, we hypothesized that the genes involved in fruit cracking in litchi might be closely related to pericarp extensibility and rapid aril expanding, so we performed cluster analysis of the DEGs in pericarp and aril via SOTA (Self-Organizing Tree Algorithm) clustering. As shown in Figure 5, the DEGs (639 genes) in pericarp were divided into seven clusters (A1–A7), of which 241 DEGs were grouped into A1 cluster, which accounts for the largest proportion, and their expression level was higher in ‘Huaizhi’ than that in ‘Nuomici’ during the whole fruit development period. A total of 180 DEGs were grouped into cluster A5 and their expression level was higher in ‘Huaizhi’ than that in ‘Nuomici’ beginning at 40 d post anthesis. In contrast, the expression level of genes in the A7 cluster was higher in ‘Nuomici’ than that in ‘Huaizhi’ beginning at 40 d post anthesis. In addition, the DEGs in cluster A4 and A6 might be less closely related to the pericarp extensibility when compared with that in other clusters since the genes in cluster A4 and A6 were expressed differentially at only one time point. Thus, DEGs (583 genes) in clusters except A4 and A6 were selected for further analysis. The DEGs (1097 genes) in aril were divided into four clusters (B1–B4). The expression of genes in cluster B1 did not change in ‘Nuomici’, but showed a higher expression level in ‘Huaizhi’. The DEGs in cluster B2 displayed a decreased expression trend in both ‘Nuomici’ and ‘Huaizhi’, but their transcripts were higher in ‘Huaizhi’ than ‘Nuomici’. The DEGs in both cluster B3 and B4 did not alter in ‘Huaizhi’, but showed a higher expression level in ‘Nuomici’. The difference is that the DEGs of cluster B3 in ‘Nuomici’ displayed a constant decline expression trend, while the expression pattern of DEGs in cluster B4 in ‘Nuomici’ was first increased and then remained unchanged.

### 2.5. Functional Annotation of Differentially Expressed Genes

**Figure 6:**
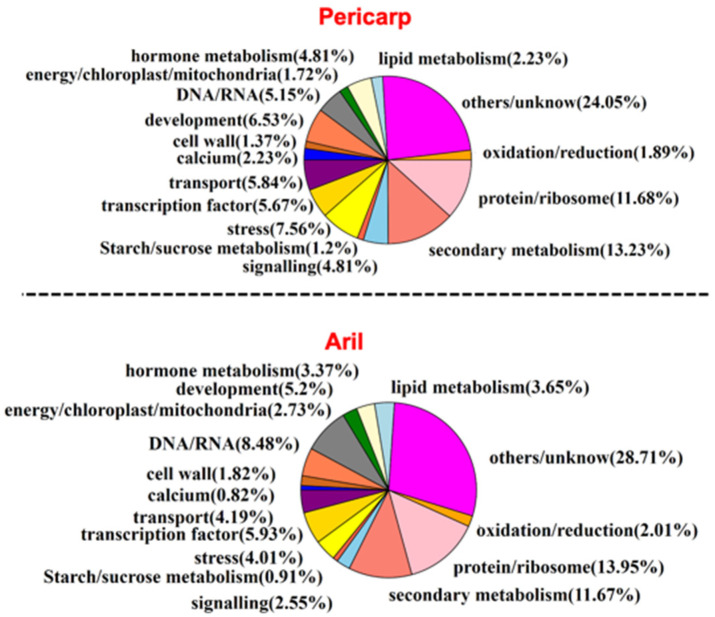
Functional annotation of DEGs with BLAST searches.

**Figure 7:**
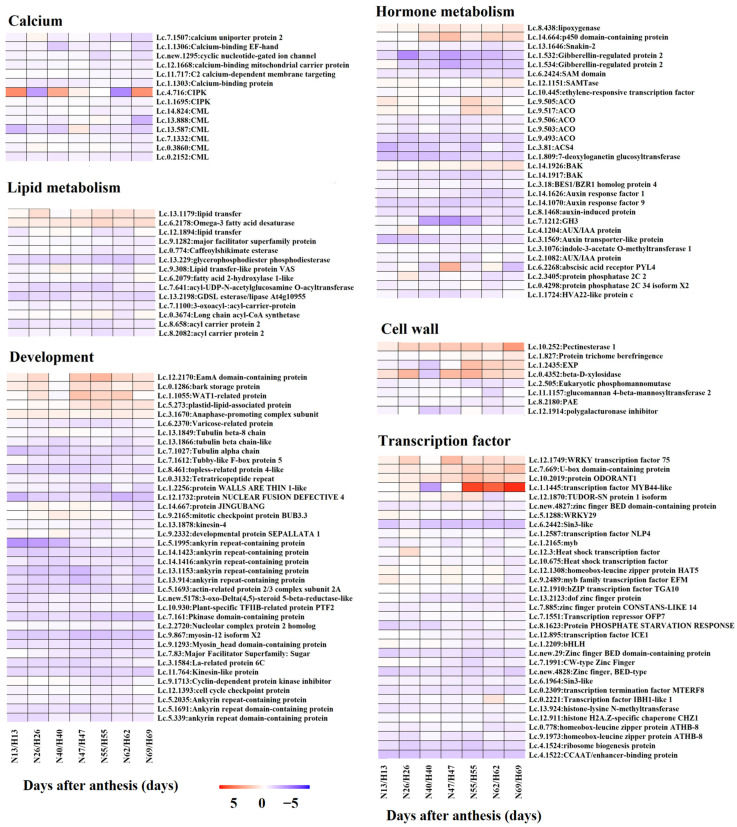
Heatmaps of DEGs in pericarp involved in specific pathways. Heatmaps showing the DEGs that are involved in calcium, lipid metabolism, development, hormone metabolism, cell wall, and transcription factors in pericarp. The ‘N’ and ‘H’ represent ‘Nuomici’ and ‘Huaizhi’, respectively. The numbers together with ‘N’ and ‘H’ indicate the days after anthesis. Fold changes in the ‘Nuomic’ relative to ‘Huaizhi’ after log2 conversion (FPKM values were converted to FPKM+1) are shown in a red-purple color scale: red, upregulated; purple, downregulated; white, unchanged.

**Figure 8:**
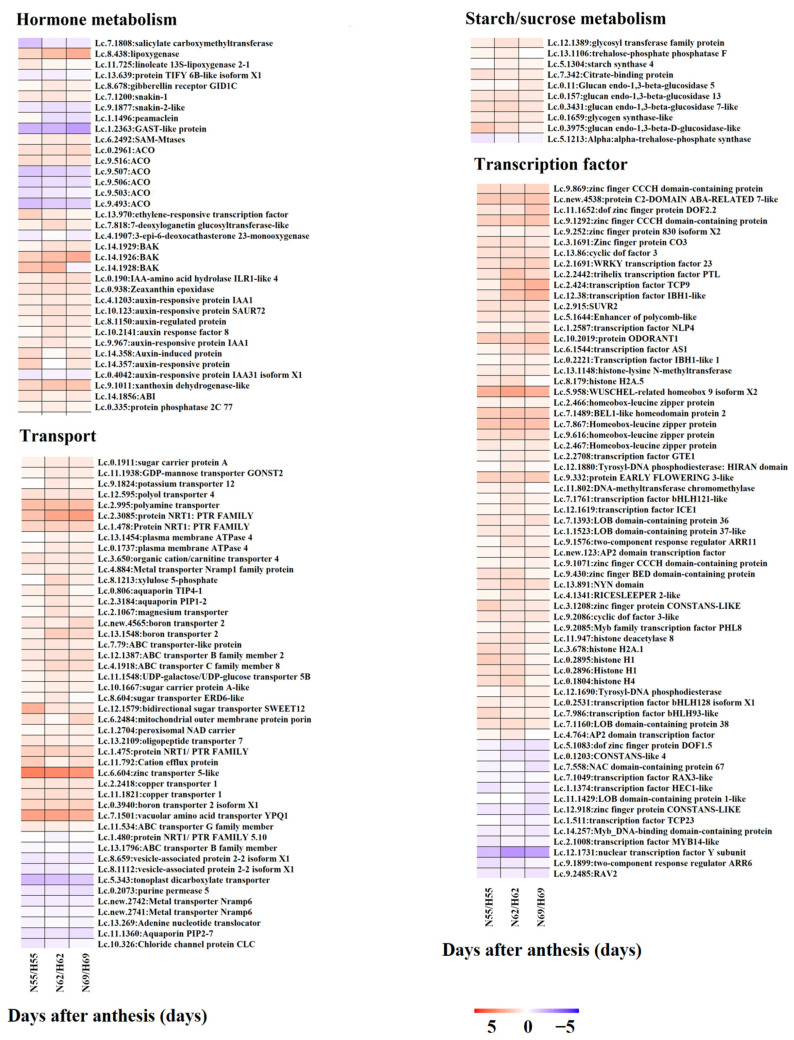
Heatmaps of DEGs in aril involved in specific pathways. Heatmaps showing the DEGs that are involved in hormone metabolism, transport, starch/sucrose metabolism, and transcription factors.

To explore the functions of DEGs in the regulation of fruit cracking in litchi, we performed functional annotation of candidate DEGs in pericarp (583 genes) and aril (1097 genes). As shown in Figure 6, both DEGs in pericarp and aril were divided into 16 groups including calcium, cell wall, development, DNA/RNA, energy/chloroplast/mitochondria, hormone metabolism, lipid metabolism, starch/sucrose metabolism, oxidation/reduction, protein/ribosome, secondary metabolism, signaling, stress, transcription factor, transporter, and others/unknown. Next, to better explore the function of these DEGs in the regulation of fruit cracking, the patterns of their expression level in ‘Nuomici’ compared with that in ‘Huaizhi’ was displayed by heatmaps. As shown in Figure 7, in pericarp, there were 14 DEGs related to calcium, all of them were significantly downregulated in ‘Nuomici’ involving calcium transport, binding, and signaling. Eight DEGs were found to be related to cell wall metabolism, of which four genes were significantly upregulated in ‘Nuomici’, involving cell wall degradation and modification. Interestingly, a total of 30 DEGs were related to hormones. Among them, 26 were significantly downregulated in ‘Nuomici’, of which eight were associated with auxin synthesis, transportation, and signal transduction. Fourteen DEGs were related to lipid metabolism. Among them, 12 DEGs were significantly downregulated in ‘Nuomici’ involving wax synthesis. Thirty-three DEGs were related to transcription factors, of which 29 DEGs were significantly downregulated in ‘Nuomici’ including the *WRKY*, *bZIP*, *bHLH*, and *MYB* transcription factors. In aril (Figure 8), there were 35 DEGs related to hormones, of which 24 were significantly upregulated in ‘Nuomici’, 10 were involved in auxin synthesis, transport, and signal transduction. Ten DEGs were related to starch/sucrose metabolism, of which nine were significantly upregulated in ‘Nuomici’ involving sucrose, β-glucoside, and trehalose transport. There were 65 DEGs that were transcription factors including *WRKY*, *bHLH*, *DOF*, *NAC*, and *MYB*. Forty-six DEGs were related to transport including 35 upregulated genes and nine downregulated genes in ‘Nuomici’ involving water, carbohydrate, and mineral elements and the transport of various small molecule compounds. Taken together, these results indicate that most of the DEGs involved in calcium, hormone metabolism, and wax metabolism were significantly downregulated in ‘Nuomici’ pericarp, while genes related to cell wall remodeling were significantly upregulated in ‘Nuomici’ pericarp. Additionally, most of the DEGs involved in hormone metabolism, starch/sucrose metabolism, and transport pathway were significantly upregulated in ‘Nuomici’ aril.

### 2.6. Validation of RNA-Seq Data

**Figure 9:**
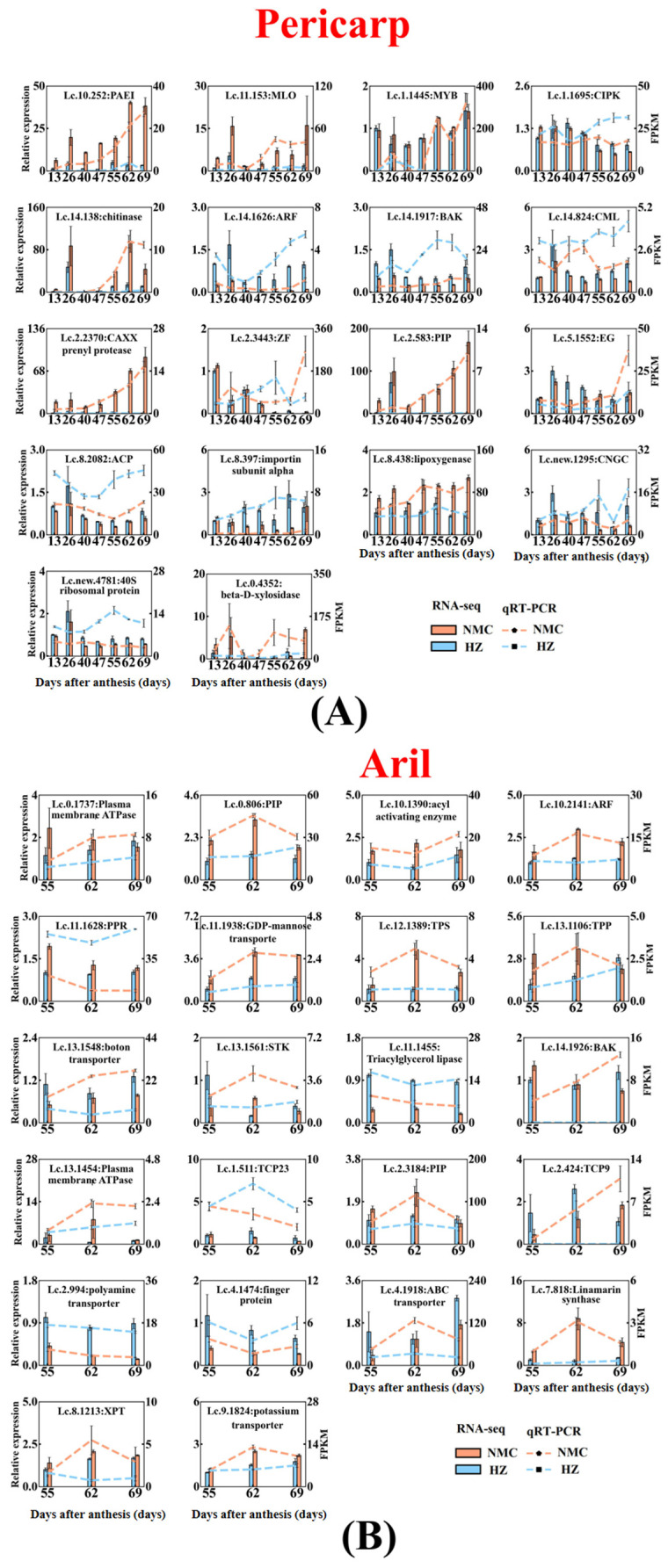
qRT-PCR (Quantitative Real-time Polymerase Chain Reaction) analysis of 40 randomly selected genes (**A**) pericarp; (**B**) aril. *Lc-EF-1α* and *Lc-Atcin* were used as reference genes for normalization of gene-expression data. Red and blue bars represent the data yielded by qRT-PCR in ‘Nuomici’ and ‘Huaizhi’, respectively. Red and blue lines represent the data yielded by RNA sequencing in ‘Nuomici’ and ‘Huaizhi’, respectively. Error bars indicate standard errors of the means (*n* = 3). The full name of each gene abbreviation is *MLO*: *Mildew resistance locus O*, *CML*: *Calmodulin-like protein*, *PIP*: *Aquaporin*, *ACP*: *Acyl carrier protein*; *ARF*: *Auxin response factor, CIPK*: *CBL-interacting serine/threonine-protein kinase, CGNC*: *Cyclic nucleotide-gated ion channel*, *PAEI*: *Pectinesterase inhibitors*, *PPR*: *Pentatricopeptide repeat-containing protein*, *BAK*: *brassinosteroid-insensitive associated receptor kinase*, *STK*: *Serine/threonine-protein kinase*, *TPS*: *Trehalose-6-phosphate synthase*, *TPP*: *Trehalose-phosphate phosphatase*, *XPT*: *Xylulose 5-phosphate translocate, EG: endo-1,4-β-glucanase*.

In order to verify the RNA-Seq data, 18 DEGs in pericarp and 22 DEGs in aril were randomly selected for qRT-PCR (Quantitative Real-time Polymerase Chain Reaction) analysis. As shown in Figure 9, the qRT-PCR results showed that the expression patterns of 12 genes in pericarp were consistent with RNA-Seq results including *PAE1*(*Pectinesterase inhibitors*, *Lc.10.252*), *MLO*(*Mildew resistance locus O*, *Lc.11.153*), *chitinase* (*Lc.14.138*), *CAXX prenyl protease* (*Lc.2.2370*), *PIP*(*Aquaporin, Lc.2.583*), *lipoxygenas* (*Lc.8.438*), and *importin subunit alpha* (*Lc.8.397*), which were upregulated in ‘Nuomici’ pericarp, and *ARF* (*Auxin response factor*, *Lc.14.1626*), *ACP*(*Acyl carrier protein*, *Lc.8.2082*), and *CNGC*(*Cyclic nucleotide-gated ion channel*, *Lc.new.1295*), which were downregulated in ‘Nuomici’ pericarp. In aril, the qRT-PCR results showed that the transcriptional patterns of 14 genes were consistent with the RNA-Seq results. Among these genes, *GDP-mannose transporter* (*Lc.11.1938*), *TPS*(*Trehalose-6-phosphate synthase, Lc.12.1389*), *TPP*(*Trehalose-phosphate phosphatase, Lc.13.1106*), *plasma membrane ATPase* (*Lc.13.1454*), *PIP* (Lc.2.3184), *linamarin synthase* (*Lc.7.818*), *potassium transporter* (*Lc.9.1824*), *PIP* (Lc.0.806), *acyl activating enzyme* (*Lc.10.1390*), and *ARF* (*Lc.10.2141*) were upregulated in ‘Nuomici’ aril, while *Triacylglycerol lipase* (*Lc.14.1455*), *TCP23* (*Lc.1.511*), *polyamine transporter* (*Lc.2.994*), and *Ring-H2 finger protein* (*Lc.4.1474*) were downregulated in ‘Nuomici’ aril.

**Figure 10:**
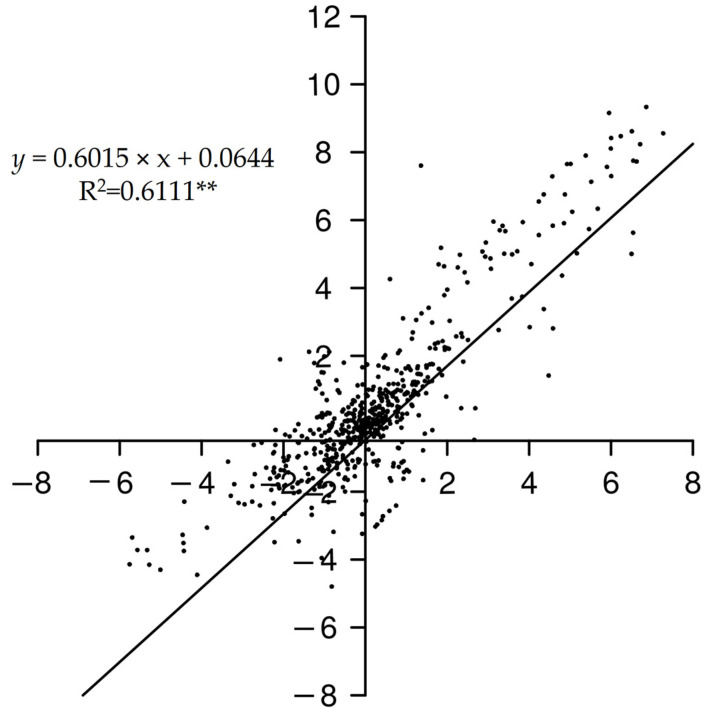
Coefficient analysis between gene expression patterns between qRT-PCR and RNA-Seq data. Extremely significant difference levels (*p* < 0.01) are indicated with two asterisks (**).

Next, the correlation coefficients between RNA-Seq and qRT-PCR were calculated (Figure 10). We found that the pairwise correlation coefficient reached a significant level higher than 0.6, indicating that expression data obtained from RNA-Seq could reflect the real transcript abundance.

## 3. Discussion

Litchi fruit cracking is a physical process of mechanical breaking. An antagonism exists between the internal aril and external pericarp, called the “ball-skin versus bladder effect”. Fruit cracking will occur upon the pressure from the internal aril expanding, which exceeds the strength from the pericarp growth.

The pericarp strength is dependent on the pericarp development status and structure. Endogenous hormones play an important role in pericarp development. Previous studies have shown that the application of NAA (Naphthylacetic acid) can reduce the fruit cracking rate in several fruit trees including litchi, citrus, sweet cherry, and apple, which may be due to the promotion of pericarp growth by NAA [24,25,26,27]. Recently, based on the analysis of metabolism in pericarp, Wang et al. pointed out that IAA deficiency in pericarp might be one main factor inducing fruit cracking in ‘Baitangying’ litchi [22]. In this study, eight auxin-related DEGs (*Lc.2.1082*: *AUX/IAA protein*, *Lc.3.1076*: *indole-3-acetate O-methyltransferase*, *Lc.3.1569*: *Auxin transporter-like protein*, *Lc.4.1204*: *AUX/IAA protein*, *Lc.7.1212*: *Gretchen Hagen3*, *Lc.8.1468*: *Auxin-induced protein*, *Lc.14.1070*: *ARF*, *Lc.14.1626*: *ARF*) were downregulated in the pericarp of cracking-susceptible cultivar ‘Nuomici’ when compared with that in cracking-resistant cultivar ‘Huaizhi’, which might lead to the retardation of pericarp growth and development in ‘Nuomici’. In addition, four genes that encode the rate-limiting enzymes for ethylene biosynthesis (*Lc.4.493*: *1-aminocyclopropane -1 -carboxlic acid synthas*, *Lc.9.493*: *l-aminocycloProPane-1-carboxylic acid oxidas*, *Lc.9.503*: *l-aminocycloProPane-1-carboxylic acid oxidas*, *Lc.9.506*: *l-aminocycloProPane-1-carboxylic acid oxidas*) and two gibberellin-regulated genes (*Lc.1.532* and *Lc.1.534*) were found to be downregulated in ‘Nuomici’ pericarp. It has been reported that ethephon and gibberellin can reduce the cracking rate of litchi [28,29]. We thus speculated that both ethylene and gibberellin might coordinate with auxin to positively regulate the development of litchi pericarp. In one hand, calcium, as an important component of cell wall to improve its mechanical strength, plays an important role in preventing pericarp cracking [30]. On one hand, calcium, as an important development and stress response [31]. Calcium sensors can transfer intracellular Ca^2+^ signals into cellular physiological responses by binding Ca^2+^ to their own EF hand binding domain. Calmodulin like protein (CML) is one of the important calcium receptors [32,33]. It has been shown that spraying calcium compounds can reduce the fruit cracking rate of sweet cherry and grape [34,35]. In litchi, one study also showed that the application of calcium chloride or calcium nitrate at the early stage of fruit development can increase the calcium concentration in litchi pericarp and reduce the fruit cracking rate [9]. In our study, we found that 14 DEGs in pericarp were related to calcium and all of them were downregulated in ‘Nuomici’ including six *calmodulin like proteins* (*Lc.0.2152*, *Lc.0.3086*, *Lc.7.1332*, *Lc.13.587*, *Lc.13.888*, *Lc.14.824*). We thus speculated that the lower expression level of calcium sensor genes might be due to lower calcium content in ‘Nuomici’ pericarp. The extensibility of pericarp is also closely related to wax deposition on the surface of pericarp [36]. *C18 fatty acyl-ACPs*, which can elongate fatty acid elongase (FAE) complexes, play important roles in the regulation of the biosynthesis of wax compounds [37]. Here, we found that the expression of two *acyl carrier protein genes* (*Lc.8.658*, *Lc.8.2082*) was significantly lower in ‘Nuomici’ pericarp than that in ‘Huaizhi’, implying that the wax component of the cell wall improved its mechanical strength and plays an important role in preventing pericarp cracking [30]. On the other hand, Ca^2+^, which can also act as a second messenger to regulate plant deposition in ‘Nuomici’ pericarp, was less than that in ‘Huaizhi’. The increased expression of cell wall remodeling genes such as *PME*, *PG*, and *EXP* can disassemble the cell wall and reduce the mechanical strength of the pericarp [38,39]. In this study, it was found that the expression level of one *expansin* (*Lc.1.2435*) and one *β-d-xylanase gene* (*Lc.0.4352*) was higher in ‘Nuomici’ pericarp than that in ‘Huaizhi’, suggesting that the strength of ‘Nuomici’ pericarp was reduced, which is consistent with previous findings that the content of cellulose, hemicellulose, and water-insoluble pectin in the pericarp of ‘Huaizhi’ was higher than that in ‘Nuomici’ [40]. In addition, we also found that most of the DEGs related to development were downregulated in ‘Nuomici’ pericarp. These genes were reported to be involved in cell division and differentiation (Figure 7). Mutation of *ACTIN-RELATED PROTEINS 2/3* in Arabidopsis results in misdirected expansion of various cell types [41]. Arabidopsis *wat1* mutants have a defect in cell elongation and display a dwarf phenotype [42]. Interestingly, these development-related genes were downregulated in ‘Nuomici’ pericarp, indicating that the development of ‘Nuomici’ pericarp may be hindered. In support of this hypothesis, Wang et al. found that the pericarp cells of the cracking-susceptible cultivar ‘Hongpinuo’ litchi stopped cell division earlier than that in the cracking-resistant cultivar ‘Baipinuo’ litchi [43].

Litchi fruit cracking is not only caused by the gradual decrease in pericarp strength, but also by the sudden increase in the pressure resulting from the aril expanding [18]. The aril pressure comes from the aril expanding, which is closely related to its osmotic potential. It has been suggested that a decrease in osmotic potential in litchi aril is associated with the degradation of macromolecules and the accumulation of assimilates in aril, and the acceleration of solute transport from other tissues into the aril [44]. In this study, we found that genes encoding one glycosyltransferase family protein (*Lc.12.1389*), four β-glucosidase *(Lc.0.11, Lc.0.3431, Lc.0.157, Lc.0.3975*), and one glycogen synthase kinase (*Lc.0.1659*) showed higher transcript abundance in ‘Nuomici’ than that in ‘Huaizhi’, implying that the accumulation of these osmotic regulation substances might be higher in the ‘Nuomici’ aril than that in the ‘Huaizhi’ aril. In addition, one potassium transporter gene (*Lc.9.1824*), one GDP-mannose transporter gene (*Lc.11.1938*), four sugar transporter genes (*Lc.8.604, Lc.10.1667, Lc.0.1911, Lc.12.1579*), and one organic cation/carnitine transporter gene (*Lc.3.650*) also showed a higher expression level in ‘Nuomici’. Previous studies have shown that the potassium transporter, GDP-mannose transporter, and organic cation/carnitine transporter (OCT) are involved in the transport of key osmotic regulation substances such as potassium, mannose, sucrose, and carnitine, respectively [45,46,47,48,49,50,51,52,53]. Taken together, these findings suggest that the osmotic potential might be lower in the ‘Nuomici’ aril than that in the ‘Huaizhi’ aril. *Aquaporin* (*PIP*) is a protein located on the cell membrane (inner membrane protein) and forms a “pore” to control water transport through the cell membrane [54]. During strawberry fruit ripening, overexpression of *PIP* can result in a fast water absorption in fruit by promoting water permeability [55]. Our RNA-Seq and qRT-PCR analysis showed that in one *PIP* gene (*Lc.2.583*), accumulation gradually increased and was higher in ‘Nuomici’ during fruit development, and showed a maximum expression difference between the ‘Nuomici’ aril and ‘Huaizhi’ aril during fruit cracking. In addition, two other *PIP* genes (*Lc.0.806, Lc.2.3184*) also had a higher expression level in the ‘Nuomici’ aril during fruit cracking. Based on these findings, we speculated that higher expression of these three PIP genes in ‘Nuomici’ might lead to stronger water absorption potential, so fruit cracking will be aggravated when fruit encounter a heavy rain [56]. The accumulation of auxin (IAA) and abscisic acid (ABA) in aril is necessary for the growth and development of the aril, but a higher content of IAA or ABA can induce fruit cracking in litchi [57]. *ILR1* could release free IAA by cleaving IAA conjugates [58]. In this study, we found that the transcript abundance of one *IAA-amino acid hydrolase* (*ILR, Lc.0.190*) was higher in the ‘Nuomici’ aril during fruit cracking than that in the ‘Huaizhi’ aril, suggesting that the level of free auxin might be higher in the ‘Nuomici’ aril than that in ‘Huaizhi’. Consistently, the transcript abundance of genes related to auxin signaling including *Auxin response protein* (*Lc.4.1203, Lc.9.967, Lc.4.1203*), *Auxin-induced protein* (*Lc.14.358*), *Small Auxin Up-Regulated genes* (*Lc.10.123*), *ARF* (*Lc.10.2141*), and *Auxin-regulated protein* (*Lc.8.1150*) was higher in the ‘Nuomici’ aril than that in ‘Huaizhi’. *Zeaxanthin epoxidase* (*ZEP*) can produce the precursor of ABA biosynthesis via catalyzing carotenoids in plant [59]. In this study, we found that one *ZEP* (*Lc.0.938*) was expressed higher in the ‘Nuomici’ aril than in the ‘Huaizhi’ aril, implying that the ABA content might be higher in the ‘Nuomici’ aril that in the ‘Huaizhi’ aril.

Transcription factors (TFs) play critical roles in the regulation of plant growth and development through modulating target genes transcription that are involved in specific plant life. To date, there is no TF that has been proven to be involved in fruit cracking directly. Here, we found that genes encoding several families of TFs such as *WRKY* (*Lc.12.1749, Lc.5.1288, Lc.2.1691*), *MYB* (*Lc.9.2085, Lc.1.2165, Lc.14.257*)*,* and *LOB* (*Lc.7.1393, Lc.1.1523, Lc.7.1160*) were differentially expressed during the fruit development and cracking between ‘Nuomici’ and ‘Huaizhi’. It is reported that overexpression of grape *vvWRKY11* in Arabidopsis can strengthen the cold resistance by adjusting the osmotic potential [60]. In strawberry, upregulation of *FaMYB44.2* can promote the accumulation of sugar and acid in fruit [61]. In banana, *MaLBDs* are involved in regulating fruit ripening through transcriptional activation of the *EXPANSIN* expression [62]. However, how these TFs are involved in fruit cracking in litchi needs to be further elucidated.

**Figure 11:**
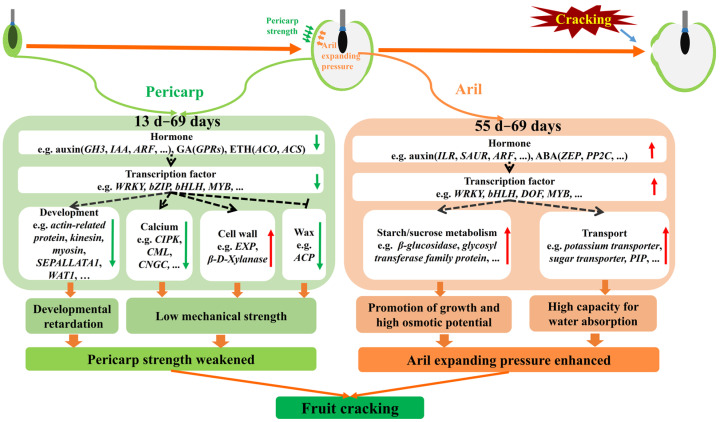
A preliminary gene network involved in litchi fruit cracking. In pericarp, the expression level of genes related to hormones IAA, GA, and ETH is low, which might directly repress the pericarp growth or indirectly downregulate specific transcription factors to repress the expression of growth-promoted genes, as a consequence, the pericarp might provide a limited space for aril expanding. In addition, the transcripts of genes related to calcium transport and signaling, and wax synthesis is also low in pericarp, while the transcript level of genes related to cell wall remodeling is high in pericarp. As a result, the pericarp of ‘Nuomici’ might be both developmental retardation and weak mechanical strength. During the aril growth, the transcripts of genes related to IAA and ABA is high, which might directly promote aril expanding or indirectly upregulate transcription factors such as *WRKY*, *bHLH*, *DOF*, *NAC*, and *MYB* to increase growth-promoted gene expression. Additionally, the transcript level of genes involved in starch/sucrose metabolism and transport is high, which could cause high osmotic potential. Notably, the expression level of one *PIP* is also high in aril, which together imply that the fruit cracking could be accelerated when fruit encounter a heavy rain since the aril might possess a strong capacity of water absorption to enhance the aril expanding pressure. Collectively, these DEGs might weaken the pericarp strength, but enhance the aril expanding pressure, thereby making the ‘Nuomici’ fruit susceptible to cracking.

Based on the expression patterns of these candidate genes and in combination with the “ball-skin versus bladder” theory, we proposed a model of molecular mechanism underlying the fruit cracking in ‘Nuomici’ (Figure 11). During the fruit growth, the transcript level of genes related to growth-promoted hormones IAA, GA and ETH (*Gretchen Hagen3: Lc.7.1212, AUX/IAA protein: Lc.4.1204, AUX/IAA protein: Lc.2.1082, Auxin-induced protein: Lc.8.1468, ARF: Lc.14.1626, gibberellin-regulated genes: Lc.1.532, gibberellin-regulated genes: Lc.1.534, 1-aminocyclopropane -1 -carboxlic acid synthas: Lc.3.81, l-aminocycloProPane-1-carboxylic acid oxidase: Lc.9.493, l-aminocycloProPane-1-carboxylic acid oxidase: Lc.9.503, l-aminocycloProPane-1-carboxylic acid oxidase: Lc.9.506*) is low in pericarp, which might directly repress the pericarp growth or indirectly downregulate transcription factors (such as *WRKY*: *Lc.5.1288*, *bZIP*: *Lc.12.1910*, *bHLH*: *Lc.1.2209*, and *MYB*: *Lc.1.2165*) to repress the pericarp growth, thereby giving a limited space for aril growth. Additionally, the transcript abundance of genes related to calcium transport and signaling (*SAMTL*: *Lc.12.1668*, *CIPK*: *Lc.4.716*, *CIPK*: *Lc.1.1695*, *CML*: *Lc.14.824*, *CML*: *Lc.13.888*, *CML*: *Lc.13.587*, *CML*: *Lc.7.1332*, *CML*: *Lc.0.3860*, *CML*: *Lc.0.2152*, *CGNC*: *Lc.new.1295*, *calcium uniporter protein*: *Lc.7.1507*), and wax synthesis (*Acyl carrier protein*: *Lc.8.658*, *Acyl carrier protein*: *Lc.8.2082*) was also low in pericarp, but the transcripts of genes related to cell wall remodeling (*EXP*: *Lc.1.2435*, *β-d-xylanase*: *Lc.0.4352*, *PME*: *Lc.10.252*) were high in pericarp, which together might weaken the mechanical strength of the ‘Nuomici’ pericarp. As a consequence, the pericarp of ‘Nuomici’ is not only developmentally retarded, but also has weak mechanical strength. During the aril growth, the expression level of genes related to growth-promoted hormones IAA (*ILR*: *Lc.0.190*, *Small Auxin Up-Regulated genes*: *Lc.10.123*, *ARF*: *Lc.0.2141*) was high in aril, which might directly promote the aril expanding or indirectly upregulate transcription factors (such as *WRKY*: *Lc.2.1691*, *bHLH*: *Lc.7.1761*, *DOF*: *Lc.9.2086*, *LOB*: *Lc.7.1393*, *LOB*: *Lc.1.1523*, *LOB*: *Lc.7.1160* and *MYB*: *Lc.9.2085*) to increase growth-promoted gene expression. Furthermore, the expression level of genes involved in starch/sucrose metabolism and transport (*β-glucosidase*: *Lc.0.11, β-glucosidase*: *Lc.0.157, β-glucosidase*: *Lc.0.3431, glycosyl transferase family*: *Lc.0.1659, potassium transporter*: *Lc.9.1824, sugar transporter*: *Lc.8.604*, *sugar transporter*: *Lc.10.1667*, *sugar transporter*: *Lc.0.1911*) was high in aril, which could enhance the osmotic potential. It is of note that the transcripts of *PIP* were also high in aril, which could promote the water transport into aril when fruit encounter a heavy rain, thereby accelerating the fruit cracking rate. Taken together, we propose that these DEGs above-mentioned could weaken the pericarp strength, but enhance the aril expanding pressure, thereby making the ‘Nuomici’ fruit susceptible to cracking.

## 4. Materials and Methods

### 4.1. Sample Collection and Data Statistics

Three 28 year-old ‘Huaizhi’ litchi trees and three 28 year-old ‘Nuomici’ litchi trees with similar initial fruit bearing were randomly selected at Xili Orchard in 2018 (Shengzhen, China). At 13 days after anthesis, twenty fruit bearing shoots located in different directions from each tree were tagged. The tagged shoots were used to monitor the fruit cracking rate and the remaining shoots were used for sample collection. Cracked fruit on tagged shoots were removed after each statistic. Pericarp samples were collected at 13, 26, 40, 47, 55, 62, and 69 days after anthesis, while aril samples were harvested beginning at 55, 62, 69 days after anthesis. All samples were immediately frozen in liquid nitrogen and stored at −80 °C for future analysis. Each tree was treated as a biological replicate.

The fruit cracking rate was indicated by the daily cracking rate [56]: (DCR, %/d) = (C1 × 00)/(C2 × T1). C1, C2, and T1 represent the number of cracked fruit, the total number of fruit, and the days between two statistics, respectively.

### 4.2. cDNA Library Construction and Illumina Sequencing

A total of 100 mg of frozen samples of pericarp or aril were ground into fine powders in liquid nitrogen and total RNA was extracted using RNAprep Pure Plant Kit (TIANGEN, Beijing, China) according to the manufacturer’s instructions. Each RNA sample was subjected to DNase digestion (TaKaRa, Dalian, China) to remove any remaining DNA. The RNA content was quantified by spectrophotometry (BioPhotometer plus, Eppendoff, Germany) and checked on 1.2% denaturing agarose gels. The RIN (RNA integrity number) values (>8.0) of these samples were assessed using an Agilent 2100 Bioanalyzer (Agilent Technologies, Santa Clara, CA, USA). The construction of the libraries and the RNA-Seq was performed by the Biomarker Biotechnology Corporation (Beijing, China). All procedures for cDNA library construction were performed via the standard Illumina sample preparation protocol. Sequencing of the purified libraries was carried out on an IlluminaHiseqTM2500 (Illumina, San Diego, CA, USA).

### 4.3. Sequence Data Analysis

Clean data were obtained by removing reads containing adapter, poly-N, and low quality reads from raw data. The Q30 and GC content of the clean data were calculated and evaluated. Clean paired end reads were mapped to the reference genome using Hisat software [63]. Read counts and normalized gene expression levels of FPKM values were obtained using Stringtie [64]. Then, all of the genes were searched against the NR, Swiss-Prot, COG, and KEGG annotation.

### 4.4. Screening of Differentially Expressed Genes (DEGs) Related to Fruit Cracking

First, co-expression network analysis was used to screen gene sets related to fruit cracking in litchi. The FPKM values between ‘Nuomici’ and ‘Huaizhi’ in pericarp or aril at the same time point were compared and DEGs were extracted according to differential expression greater than two-fold (FDR < 0.01, log_2_ > 1 or < −1). After that, the DEGs in pericarp at each time point were combined and defined as P1, and DEGs in aril were defined as A1. Then, the genes in P1 and A1 were divided into different modules using WGCNA with a default parameter setting, respectively. Finally, the WGCNA package was used to calculate the correlation and significance among different gene modules and DCR. The DEGs in modules of pericarp and aril that had significant correlation with DCR were defined as PM and AM, respectively. Meanwhile, the DEGs between ‘Nuomici’ and ‘Huaizhi’ in pericarp and aril during fruit cracking were also screened and recorded as P2 and A2, respectively. Next, the DEGs shared by AM and A2, PM and P2 were identified as candidate genes that are involved in the fruit cracking of ‘Nuomici’.

In addition, the SOTA cluster analysis was performed on the DEGs shared by AM and A2, PM and P2 using MeV software (http://mev.tm4.org), TreeView (http://jtreeview.sourceforge.net), which was used to visualize the clustering of genes [65,66].

### 4.5. Quantitative RT-PCR (qRT-PCR) Validation

RNA was reverse-transcribed into cDNA by TransScript^®^ One-Step gDNA Removal and cDNA Synthesis SuperMix (TransGen, Beijing, China) with gDNA Remover. Q-PCR was performed on a LightCycler 480II (Roche, Basel, Switzerland) using the SYBR Green Realtime PCR Master Mix (TOYOBO, Osaka, Japan) as the readout. The running parameters of the PCR amplifications included the following conditions: 95 °C for denaturation at 30 s, followed by 40 cycles of denaturation at 95 °C for 5 s, annealing at 60 °C for 30 s, and extension at 72 °C for 30 s. Then, PCR products were analyzed by the melting curve to confirm the specificity of amplification. Primers were designed using Primer5 and are listed in Table S1. In this study, *LcEF1α*-normalized expressions are presented in Figure 9, which were reproducible after *LcActin* normalization [67]. Relative gene expression levels were calculated using the 2^−^^△△CT^ method with three independent biological replicates [68]. Primers used here are listed in Table S1 and the qPCR melting curves are shown in Figure S1.

### 4.6. Data Deposition

All the raw read sequences were deposited in the NCBI (National Center for Biotechnology Information) sequence read archive under BioProject accession PRJNA681070.

## Figures and Tables

**Table 1:** Statistical results of the output data and mapping rate.

Category -	Pericarp	Aril
Clean reads	2,763,324,022	473,002,306
GC content (%)	45.81	46.42
Q30 percent (%)	94.01	94.03
Mapping rate (%)	79.68	78.72

Q30 percent: The percentages of the bases whose Phred values were more than 30; GC content: The content of guanine and cytosine for the library in one tissue.

## Data Availability

The raw data of the RNA-Seq have been submitted to NCBI Sequence Read Archive (SRA) under BioProject accession PRJNA681070.

## Supplementary Materials

The following are available online at https://www.mdpi.com/1422-0067/22/1/454/s1, Figure S1: Melt-curves and Melt-Peak for the primers used in this study, Table S1: Primers for qRT-PCR analysis in this study.

## Abbreviations

WGCNA	weighted gene co-expression network analysis
IAA	indole-3-acetic acid
GA	gibberellins
ETH	ethylene
ABA	abscisic acid
EXP	expansin
EG	endo-1,4-β-glucanase
PG	polygalacturonase
XET	xyloglucan endotrans-glycosylase
TAG	glycerolipid triacylglycerol
MDP	MADS box transcription factors
RNAi	RNA interference
DEGs	differentially expressed genes
DCR	daily cracking rate
ARF	Auxin response factor
CIPK	CBL-interacting serine/threonine-protein kinase
CGNC	Cyclic nucleotide-gated ion channel
PAEI	Pectinesterase inhibitors
PPR	Pentatricopeptide repeat-containing protein
BAK	Brassinosteroid-insensitive associated receptor kinase
STK	Serine/threonine-protein kinase
TPS	Trehalose-6-phosphate synthase
TPP	Trehalose-phosphate phosphatase
XPT	Xylulose 5-phosphate translocator
NCBI	National Center for Biotechnology Information
FPKM	Fragments Per Kilobase Million
FDR	False discovery rate
SOTA	Self-Organizing Tree Algorithm
qRT-PCR	Quantitative Real-time Polymerase Chain Reaction
MLO	Mildew resistance locus O
CML	Calmodulin-like protein
PIP	Aquaporin
ACP	Acyl carrier protein
TCP	TEOSINTE BRANCHED 1, CYCLOIDEA, PCF1
NAA	Naphthylacetic acid
PME	Pectin methylesterases

## References

[1] Correia S., Schouten R., Silva A., Gonçalves B. (2018). Sweet cherry fruit cracking mechanisms and prevention strategies: A review. Sci. Hortic..

[2] Opara L.U., Hodson A.D., Studman S.P. (2000). Stem-end splitting and internal ring-cracking of ‘*Gala*’ apples as influenced by orchard management practices. J. Hortic. Sci. Biotechnol..

[3] Li J., Chen J.Z. (2017). Citrus Fruit-Cracking: Causes and Occurrence. Hortic. Plant J..

[4] Gibert C., Genard M., Vercambre G., Lescourret F. (2010). Quantification and modelling of the stomatal, cuticular and crack components of peach fruit surface conductance. Funct. Plant Biol..

[5] Considine J.A. (1982). Physical Aspects of Fruit Growth: Cuticular Fracture and Fracture Patterns in Relation to Fruit Structure in *Vitis Vinifera*. J. Hort. Sci..

[6] Khadivi-Khub A. (2014). Physiological and genetic factors influencing fruit cracking. Acta Physiol. Plant.

[7] Davarpanah S., Tehranifar A., Abadía J., Val J., Davarynejad G., Aran M., Khorassani R. (2018). Foliar calcium fertilization reduces fruit cracking in pomegranate (*Punica granatum* cv. Ardestani). Sci. Hortic..

[8] Alvarez-Herrera J., Balaguera-López H., Fischer G. (2012). Effect of Irrigation and Nutrition with Calcium on Fruit Cracking of the Cape Gooseberry (*Physalis peruviana* L.) in the Three Strata of the Plant. Acta Hortic..

[9] Haq I.U., Rab A. (2012). Characterization of physico-chemical attributes of litchi fruit and its relation with fruit skin cracking. J. Anim. Plant Sci..

[10] Peña Estevez M., Artés-Hernández F., Aguayo E., Martínez-Hernández G., Galindo A., Artés F., Gómez P. (2013). Effect of sustained deficit irrigation on physicochemical properties, bioactive compounds and postharvest life of pomegranate fruit (cv.‘Mollar de Elche’). Postharvest Biol. Technol..

[11] Cuartero J., Palomares G., Balasch S., Nuez F. (1981). Tomato fruit cracking under plastic-house and in the open air. II. General and specific combining abilities. BMC Infect. Dis..

[12] Kasai S., Hayama H., Kashimura Y., Kudo S., Osanai Y. (2008). Relationship between fruit cracking and expression of the expansin gene *MdEXPA3* in ‘Fuji’ apples (*Malus domestica* Borkh.). Sci. Hortic..

[13] Balbontin C., Ayala H., Rubilar J., Cote J., Figueroa C.R. (2014). Transcriptional analysis of cell wall and cuticle related genes during fruit development of two sweet cherry cultivars with contrasting levels of cracking tolerance. Chil. J. Agric. Res..

[14] Jiang H.K., Tian H.M., Yan C.S., Jia L., Wang Y., Wang M.X., Jiang C.J., Li Y.Y., Jiang J.Y., Fang L. (2019). RNA-seq analysis of watermelon (*Citrullus lanatus*) to identify genes involved in fruit cracking. Sci. Hortic..

[15] Jiang F., Lopez A., Jeon S., De Freitas S.T., Yu Q.H., Wu Z., Labavitch J.M., Tian S.K., Powell A.L.T., Mitcham E. (2019). Disassembly of the fruit cell wall by the ripening-associated *polygalacturonase* and *expansin* influences tomato cracking. Hortic. Res..

[16] Li J.G., Huang H.B., Huang X.M. (2003). A Revised Division of the Developmental Stages in Litchi Fruit. Acta Horticulturae Sinica.

[17] Huang H.B., Xu J.K. (1983). The developmental patterns of fruit tissue and their correlative relationships in *Litchi chinensis* Sonn. Sci. Hortic..

[18] Li J.G., Huang H.B., Gao F.F., Huang X.M., Wang H.C. (2001). An overview of litchi fruit cracking. Acta Hortic..

[19] Lu W., Wang Y., Jiang Y., Li J., Liu H., Duan X., Song L. (2006). Differential expression of litchi *XET* genes in relation to fruit growth. Plant Physiol. Biochem..

[20] Wu F.W., Kuang J.F., Lu W.J., Liu H.J., Cheng J.Y. (2009). Cloning and Expression Analysis of *EG* Genes in Litchi Fruit. Acta Hortic. Sin..

[21] Li W.C., Wu J.Y., Zhang H.N., Shi S.Y., Liu L.Q., Shu B., Liang Q.Z., Xie J.H., Wei Y.Z. (2014). *De Novo* Assembly and Characterization of Pericarp Transcriptome and Identification of Candidate Genes Mediating Fruit Cracking in *Litchi chinensis* Sonn. Int. J. Mol. Sci..

[22] Wang J.G., Gao X.M., Ma Z.L., Chen J., Liu Y.N., Shi W.Q. (2019). Metabolomic and transcriptomic profiling of three types of litchi pericarps reveals that changes in the hormone balance constitute the molecular basis of the fruit cracking susceptibility of *Litchi chinensis* cv. Baitangying. Mol. Biol. Rep..

[23] Langfelder P., Horvath S. (2008). WGCNA: An R package for weighted correlation network analysis. BMC Bioinformatics.

[24] Kumar K., Pinder R., Dabas S.T.K., Yadav B., Rana S. (2017). Effect of growth regulators and micronutrients on fruit cracking and fruit yield in pomegranate. Indian J. Agric. Res..

[25] Greenberg J., Kaplan I., Fainzack M., Egozi Y., Giladi B. (2006). Effects of Auxins Sprays on Yield, Fruit Size, Fruit Splitting and the Incidence of Creasing of ‘Nova’ mandarin. Acta Hortic..

[26] Wang Y., Lu W.J., Li J.G., Jiang Y.M. (2006). Differential Expression of Two *Expansin* Genes in Developing Fruit of Cracking-susceptible and -resistant Litchi Cultivars. J. Am. Soc. Hortic. Sci..

[27] Le N.V., Yen C.R., Tsai S.H. (2017). Influence of naphthalene acetic acid (NAA) on yield and quality of wax apple (*Syzygium samaragense*). Acta Hortic..

[28] Srivastava R.P., Singh L. (1969). Effect of growth substances on the quality of litchi. Hort. Sci..

[29] Sharma S.B., Dhillon B.S. (1984). Effect of zinc sulphate and growth regulators on the growth of litchi fruit. Prog. Hortic..

[30] Jarvis M., Briggs S., Knox P. (2003). Intercellular adhesion and cell separation in plants. Plant Cell Environ..

[31] Tuteja N., Mahajan S. (2007). Calcium Signaling Network in Plants: An Overview. Plant Signal. Behav..

[32] Edel K., Marchadier E., Brownlee C., Kudla J., Hetherington A. (2017). The Evolution of Calcium-Based Signalling in Plants. Curr. Biol..

[33] Ding X.C., Zhang L.P., Hao Y.W., Xiao S.L., Wu Z., Chen W.X., Li X.P., Zhu X.Y. (2018). Genome-wide identification and expression analyses of the calmodulin and calmodulin-like proteins reveal their involvement in stress response and fruit ripening in papaya. Postharvest Biol. Technol..

[34] Yu J., Zhu M.T., Bai M., Xu Y.S., Fan S.G., Yang G.S. (2020). Effect of calcium on relieving berry cracking in grape (*Vitis vinifera* L.) ‘Xiangfei’. PeerJ.

[35] Brown G., Wilson S., Boucher W., Graham B., McGlasson B. (1995). Effects of copper-calcium sprays on fruit cracking in sweet cherry (*Prunus avium*). Sci. Hortic..

[36] Leide J., Hildebrandt U., Reussing K., Riederer M., Vogg G. (2007). The Developmental Pattern of Tomato Fruit Wax Accumulation and Its Impact on Cuticular Transpiration Barrier Properties: Effects of a Deficiency in a β -Ketoacyl-Coenzyme A Synthase (LeCER6). Plant Physiol..

[37] Yeats T., Rose J. (2013). The Formation and Function of Plant Cuticles. Plant Physiol..

[38] Cantu D., Vicente A., Greve L.C., Dewey F., Bennett A., Labavitch J.M., Powell A. (2008). The intersection between cell wall disassembly, ripening, and fruit susceptibility to Botrytis cinerea. PNAS.

[39] Darley C., Forrester A., McQueen-Mason S. (2001). The molecular basis of plant cell extension. Plant Mol. Biol..

[40] Huang X.M., Wang H.C., Gao F.F., Huang H.B. (1999). A comparative study of the pericarp of litchi cultivars susceptible and resistant to fruit cracking. J. Hortic. Sci..

[41] Mathur J., Mathur N., Kernebeck B., Hülskamp M. (2003). Mutations in Actin-Related Proteins 2 and 3 Affect Cell Shape Development in Arabidopsis. Plant Cell.

[42] Ranocha P., Denancé N., Vanholme R., Freydier A., Martinez Y., Hoffmann L., Köhler L., Pouzet C., Renou J.-P., Sundberg B. (2010). Walls are thin 1 (WAT1), an Arabidopsis homolog of *Medicago truncatula NODULIN21*, is a tonoplast-localized protein required for secondary wall formation in fibers. Plant J..

[43] Wang H.C., Wei B.W., Gao F.F. (2000). Studies on the relation among fruit skin structure, cell division and fruit cracking in litchi (*Litchi chinensis* Sonn.). J. South China Agri. Univ..

[44] Li J.G., Huang H.B. (1996). Advance in the research in litchi fruit cracking. J. Fruit Sci..

[45] Wang S.F., Song M.Y., Guo J.X., Huang Y., Zhang F., Xu C., Xiao Y.H., Zhang L.S. (2017). The potassium channel *FaTPK1* plays a critical role in fruit quality formation in strawberry (*Fragaria × ananassa*). Plant Biotechnol. J..

[46] Ahmad I., Devonshire J., Mohamed R., Schultze M., Maathuis F. (2015). Overexpression of the potassium channel TPKb in small vacuoles confers osmotic and drought tolerance to rice. New Phytol..

[47] Yu Z.M., He C.M., Teixeira da Silva J., Luo J.P., Yang Z.Y., Duan J. (2018). The GDP-mannose transporter gene (*DoGMT*) from *Dendrobium officinale* is critical for mannan biosynthesis in plant growth and development. Plant Sci..

[48] Chen L.Q. (2014). SWEET sugar transporters for phloem transport and pathogen nutrition. New Phytol..

[49] Liu R.L., Li B.Q., Qin B.Q., Zhang Z.Q., Tian S.P. (2017). Identification and Functional Characterization of a Tonoplast Dicarboxylate Transporter in Tomato (*Solanum lycopersicum*). Front. Plant Sci..

[50] Zahoor R., Zhan W.Z., Abid M., Dong H.R., Zhou Z.G. (2017). Title: Potassium application regulates nitrogen metabolism and osmotic adjustment in cotton (*Gossypium hirsutum* L.) functional leaf under drought stress. J. Plant Physiol..

[51] Li Z., Yong B., Cheng B.Z., Wu X., Zhang Y., Zhang X.Q., Peng Y. (2019). Nitric oxide, γ-aminobutyric acid, and mannose pretreatment influence metabolic profiles in white clover under water stress. J. Integr. Plant Biol..

[52] Ahmadi A., Baker D.A. (2001). The effect of water stress on the activities of key regulatory enzymes of the sucrose to starch pathway in wheat. Plant Growth Regul..

[53] Charrier A., Rippa S., Yu A., Nguyen P.J., Renou J.P., Perrin Y. (2011). The effect of carnitine on Arabidopsis development and recovery in salt stress conditions. Planta.

[54] Chaumont F., Tyerman S. (2014). Aquaporins: Highly Regulated Channels Controlling Plant Water Relations. Plant Physiol..

[55] Mut P., Bustamante C., Martínez G., Alleva K., Sutka M., Civello M., Amodeo G. (2008). A fruit-specific plasma membrane aquaporin subtype PIP1;1 is regulated during strawberry (*Fragaria × ananassa*) fruit ripening. Physiol Plant..

[56] Li J.G., Huang H.B., Yuan R.C., Gao F.F. (1992). Litchi fruit cracking in relation to fruit growth and water-uptake kinetics. J. South China Agri. Univ..

[57] Marboh E., Singh S.K., Swapnil P., Nath V., Gupta A.K., Pongener A. (2017). Fruit cracking in litchi (*Litchi chinensis*): An overview. Indian J. Agric. Res..

[58] Leclere S., Tellez R., Rampey R., Matsuda S., Bartel B. (2002). Characterization of a Family of IAA-Amino Acid Conjugate Hydrolases from *Arabidopsis*. J Biol. Chem..

[59] Seo M., Koshiba T. (2002). Complex regulation of ABA biosynthesis in plants. Trends Plant. Sci..

[60] Liu H.Y., Yang W.L., Liu D.C., Han Y.P., Zhang A.M., Li S.H. (2011). Ectopic expression of a grapevine transcription factor *VvWRKY11* contributes to osmotic stress tolerance in *Arabidopsis*. Mol. Biol. Rep..

[61] Wei L.Z., Mao W.W., Jia M.R., Xing S.N., Ali U., Zhao Y., Chen Y., Cao M., Dai Z., Zhang K. (2018). FaMYB44.2, a transcriptional repressor, negatively regulates sucrose accumulation in strawberry receptacles through interplay with FaMYB10. J. Exp. Bot..

[62] Ba L.J., Shan W., Kuang J.F., Feng B.H., Xiao Y.Y., Lu W.J., Chen J.Y. (2014). The banana MaLBD (lateral organ boundaries domain) transcription factors regulate *EXPANSIN* expression and are involved in fruit ripening. Plant Mol. Biol. Report..

[63] Pertea M., Kim D., Pertea G., Leek J., Salzberg S. (2016). Transcript-level expression analysis of RNA-seq experiments with HISAT, StringTie and Ballgown. Nat. Protoc..

[64] Pertea M., Pertea G., Antonescu C., Chang T.-C., Salzberg S. (2015). StringTie enables improved reconstruction of a transcriptome from RNA-seq reads. Nat. Biotechnol..

[65] Howe E., Sinha R., Schlauch D., Quackenbush J. (2011). RNA-Seq analysis in MeV. Bioinformatics.

[66] Saldanha A.J. (2004). Java Treeview—extensible visualization of microarray data. Bioinformatics.

[67] Zhong H.Y., Chen J.W., Li C.Q., Chen L., Wu J.Y., Chen J.Y., Lu W.J., Li J.G. (2011). Selection of reliable reference genes for expression studies by reverse transcription quantitative real-time PCR in litchi under different experimental conditions. Plant Cell Rep..

[68] Livak K.J., Schmittgen T.D. (2002). Analysis of Relative Gene Expression Data Using Real-Time Quantitative PCR and the 2^−ΔΔCT^ Method. Methods.

