# Supplementary material for: RNA-Seq Provides New Insights into the Molecular Events Involved in “Ball-Skin versus Bladder Effect” on Fruit Cracking in Litchi
Source: Int J Mol Sci. 2021 Jan 5;22(1):454. doi: 10.3390/ijms22010454 (PMC7796454; doi:10.3390/ijms22010454)
Supplement: Supplementary file 1 [file ijms-22-00454-s001.zip › Figure S1.docx]

**Figure S1. Melt-curves and Melt-Peak for the primers used in this study**

***Lc.10.252***


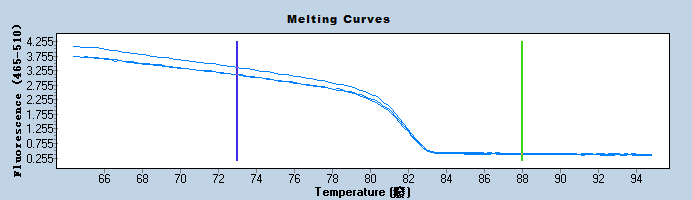

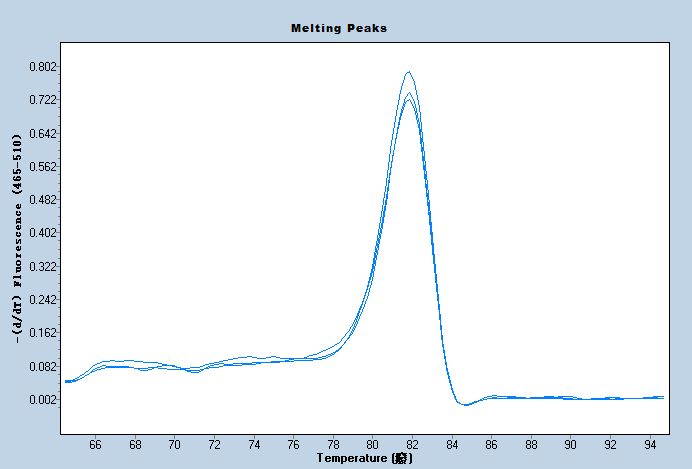


***Lc.11.153***


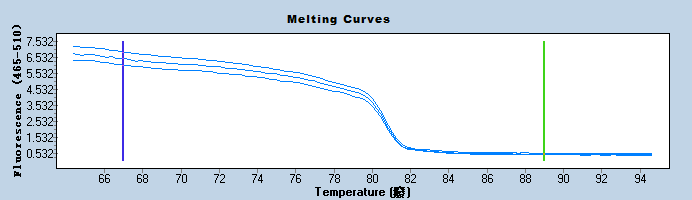

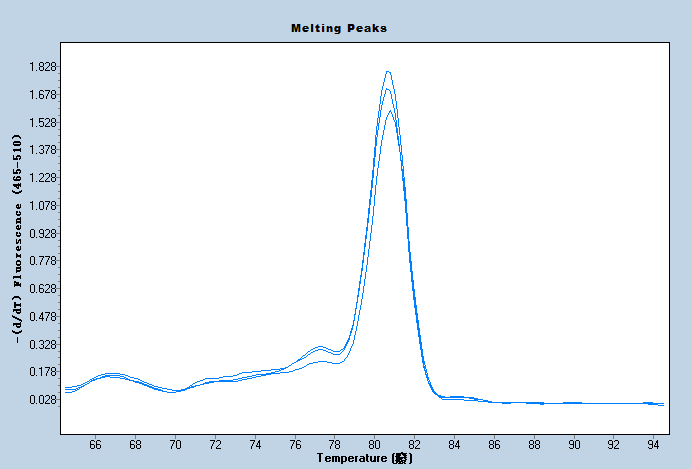


***Lc1.1445***

**
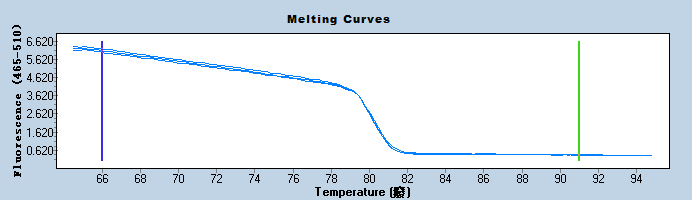
** ***
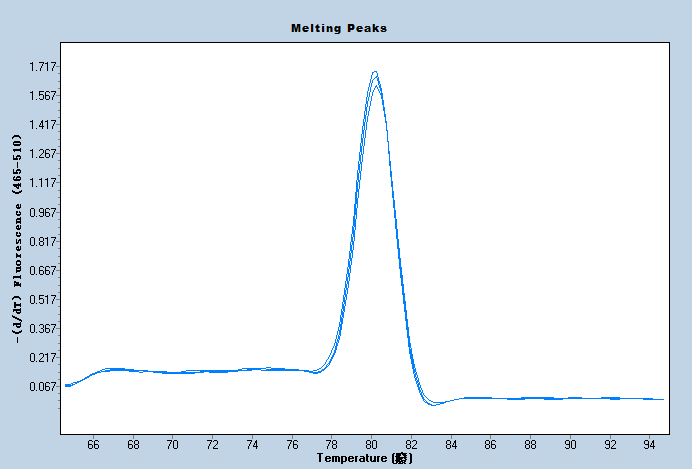
***

***Lc.1.1695***


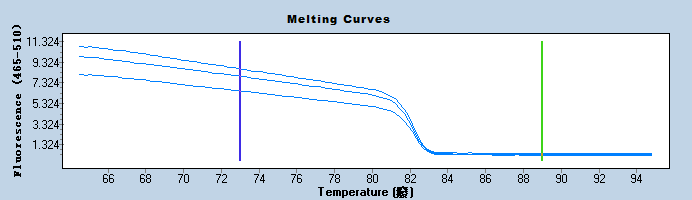

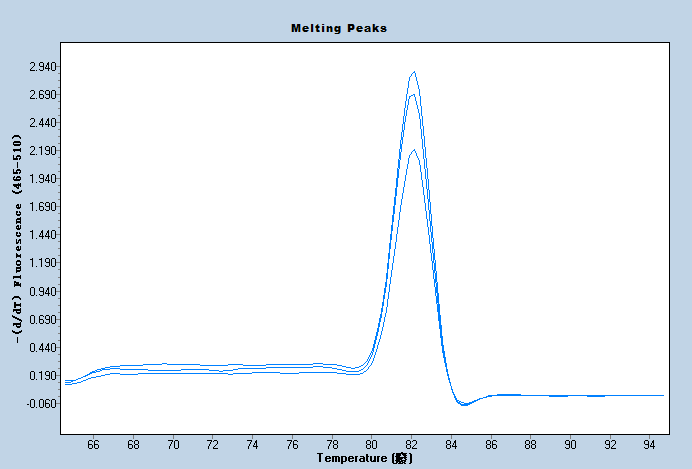


***Lc.14.138***


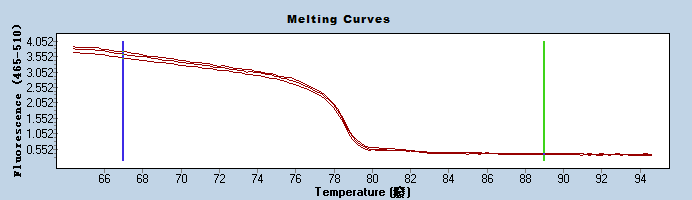

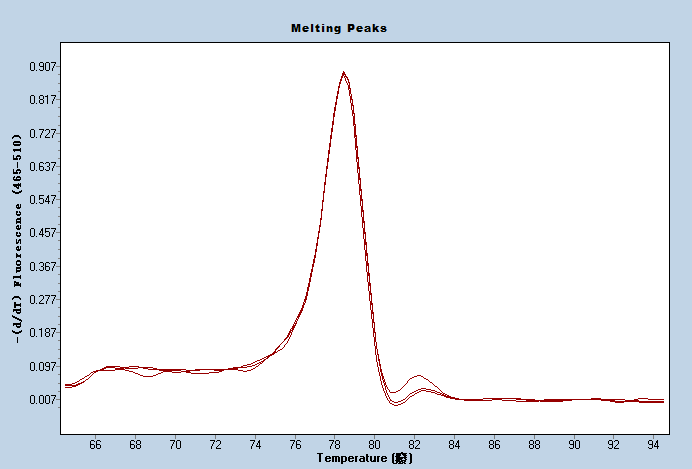


***Lc.14.1626***


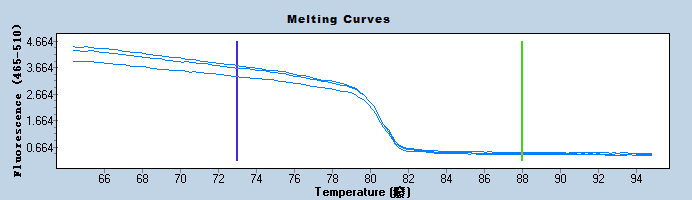

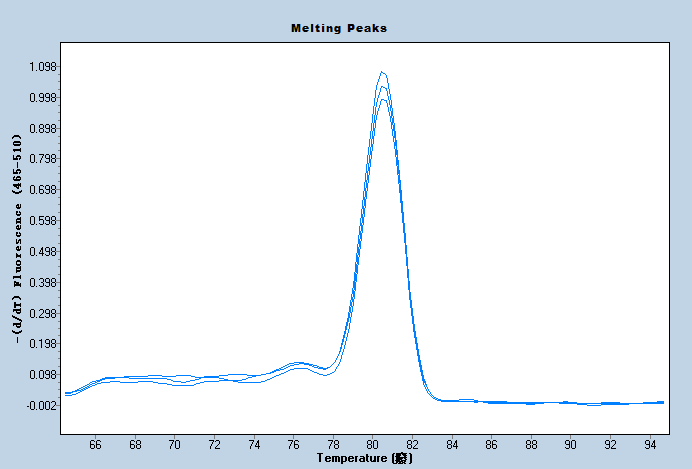


***Lc.14.1917***


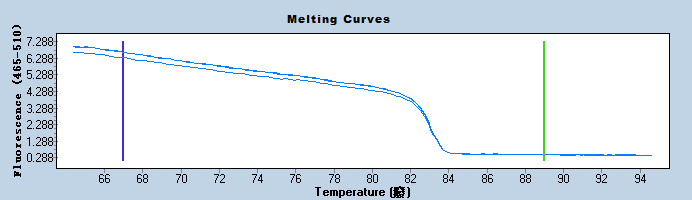

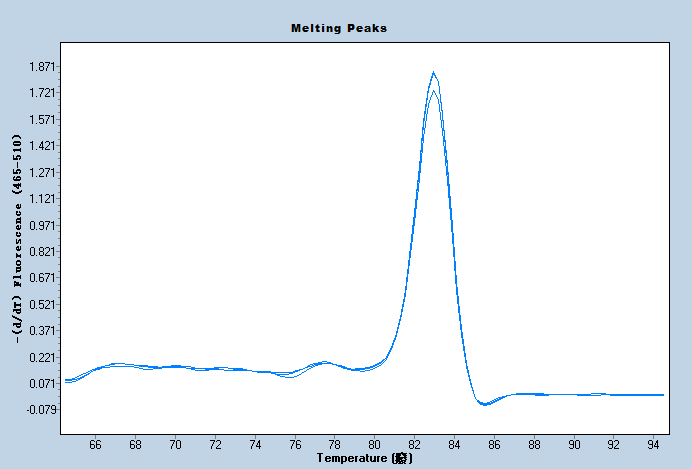


***Lc.14.824***

**
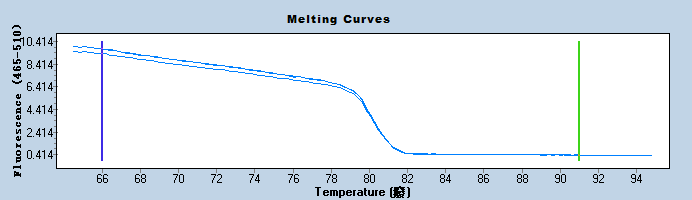
*
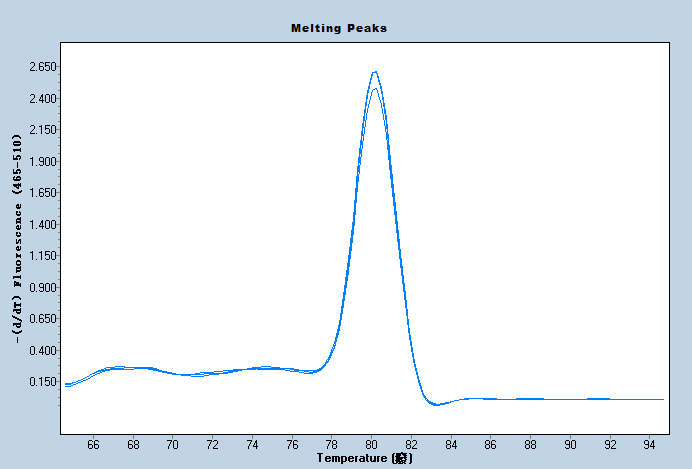
***

***Lc.2.2370***


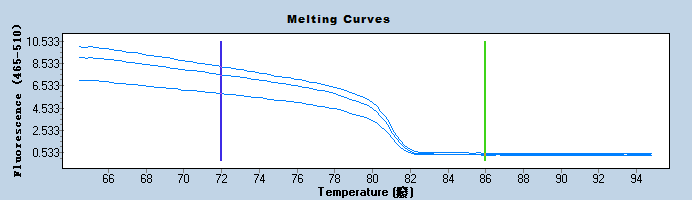

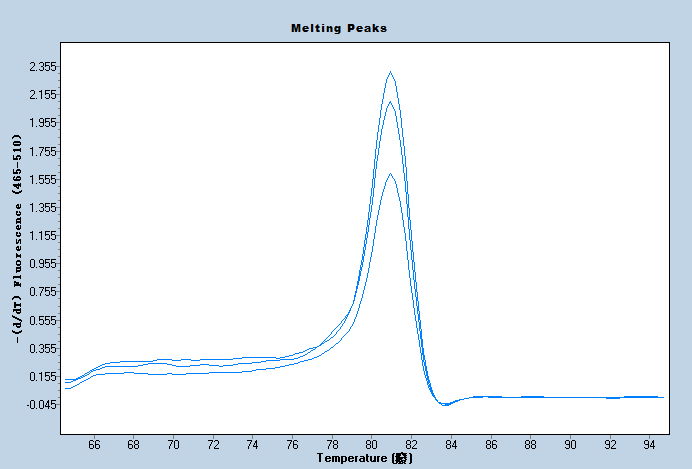


***Lc.2.3443***


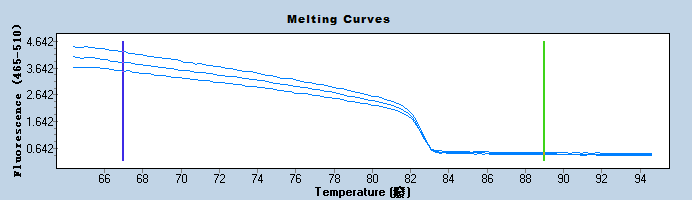

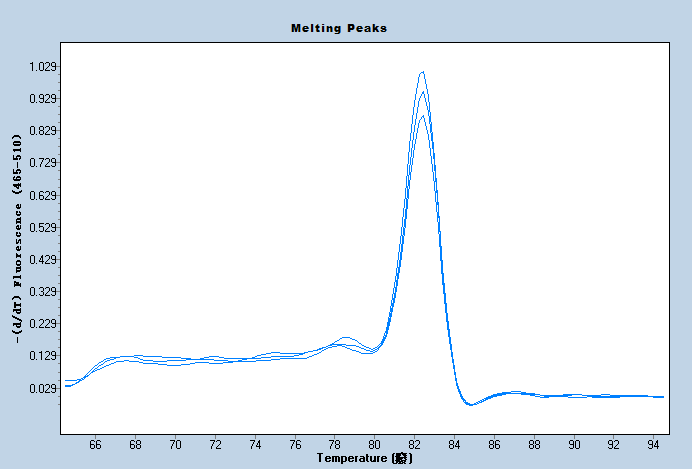


***Lc.2.583***


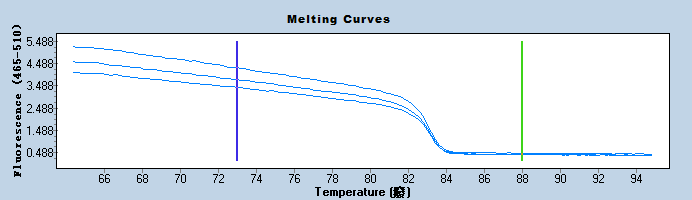
***
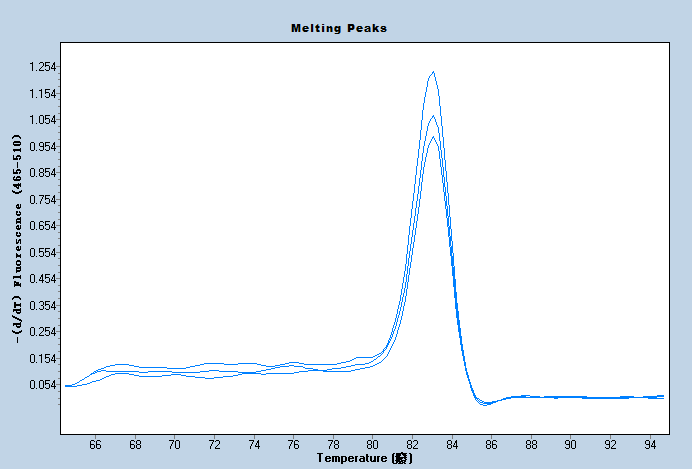
***

***Lc.5.1552***


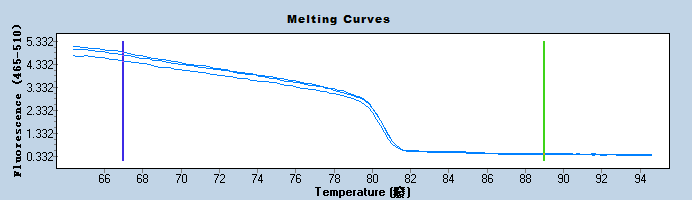

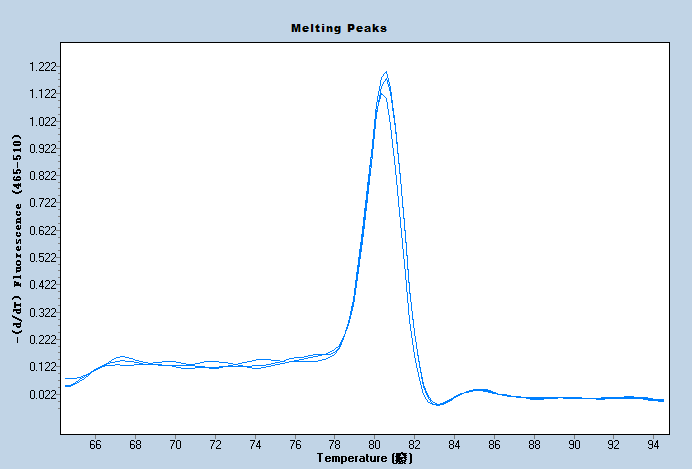


***Lc.8.2082***


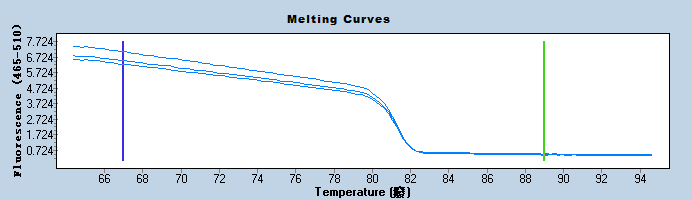

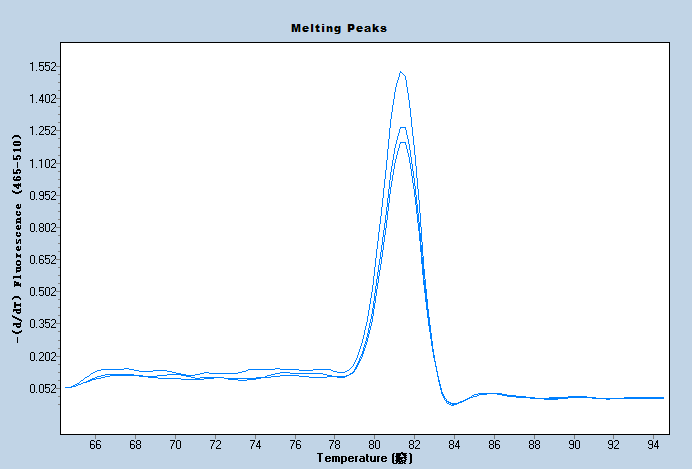


***Lc.8.397***

**
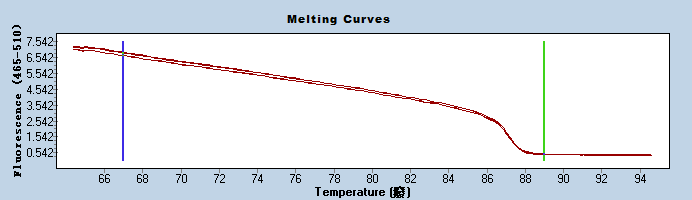
*
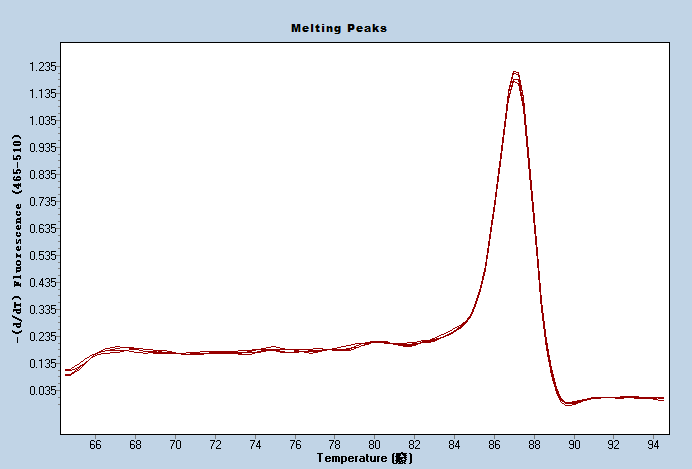
***

***Lc.8.438***

**
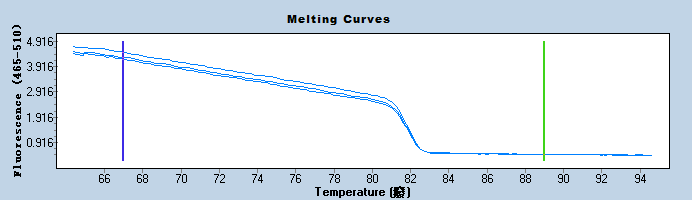
*
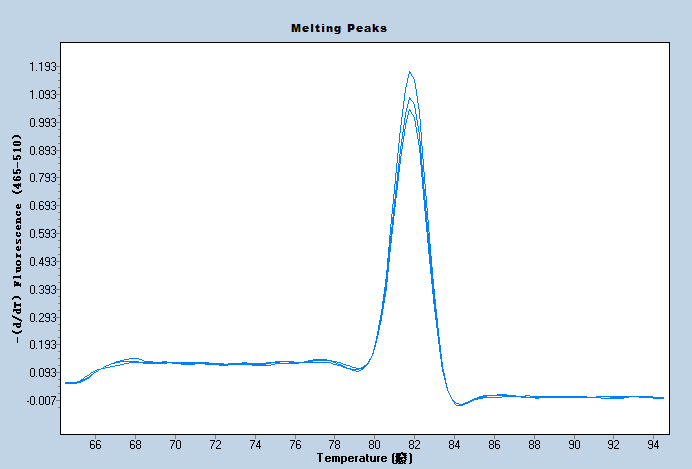
***

***Lc.new.1295***


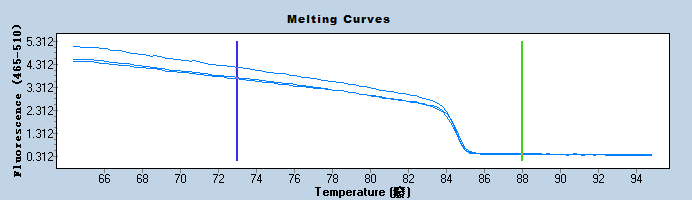

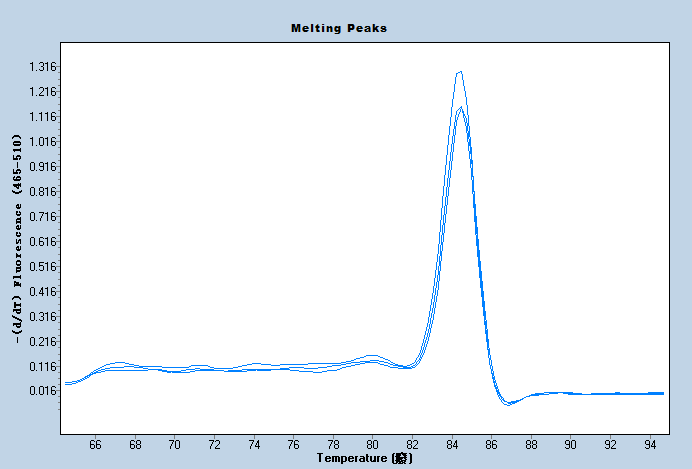


***Lc.new.4781***

***
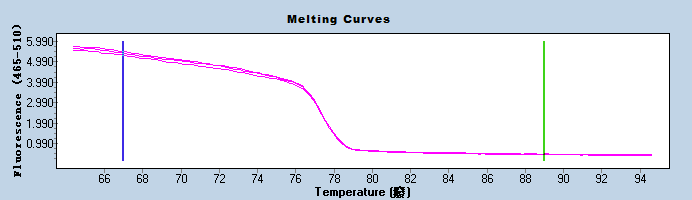

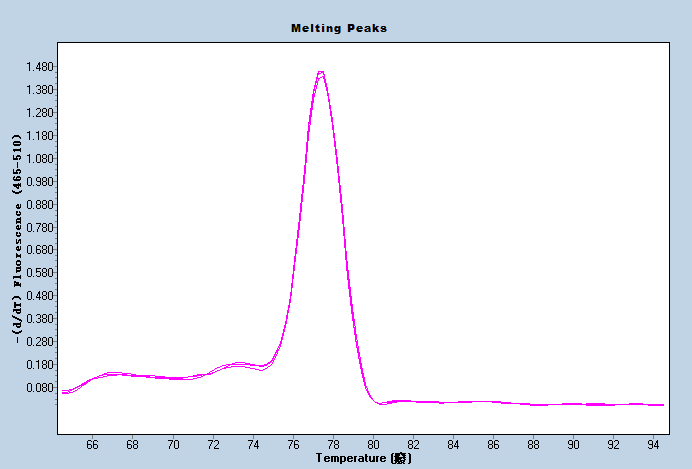
***

***Lc.0.4352***


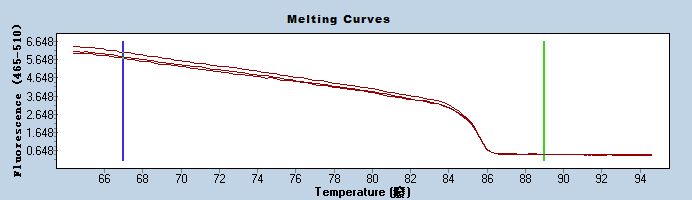

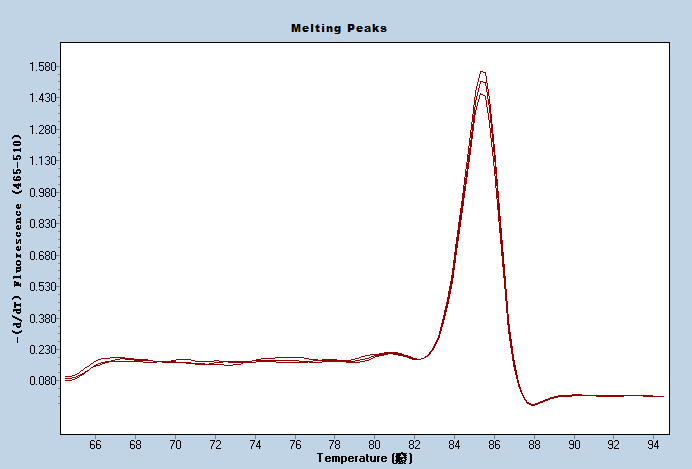


***Lc.0.1737***


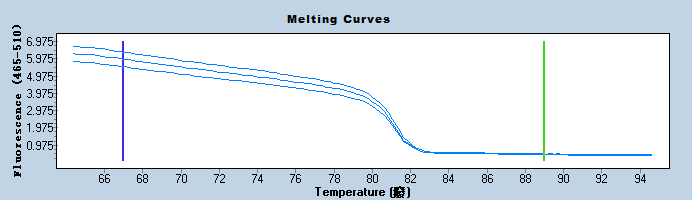

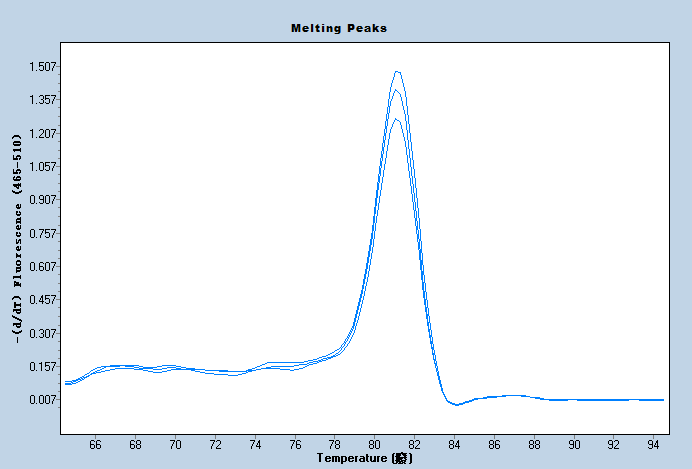


***Lc.0.806***

***
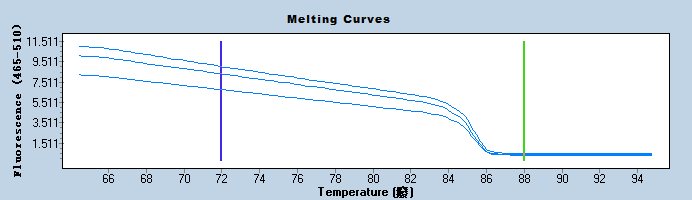
*** ***
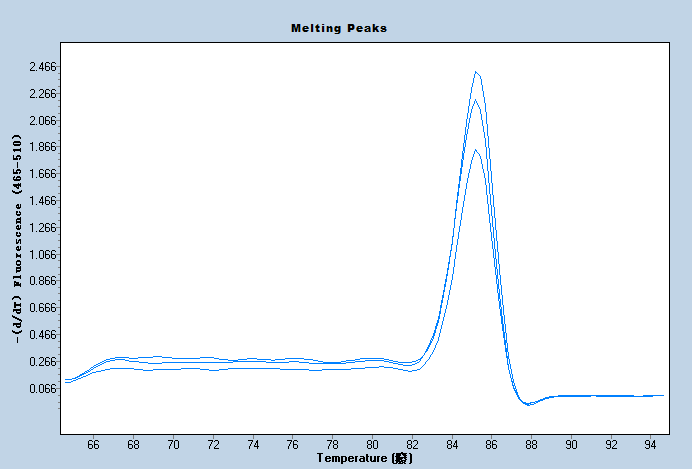
***

***Lc.10.1390***


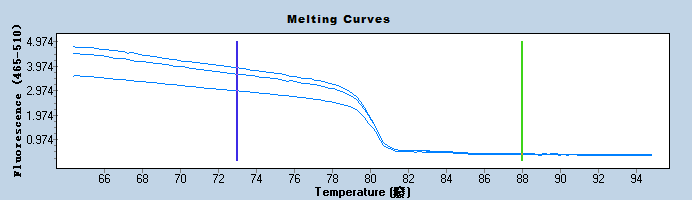

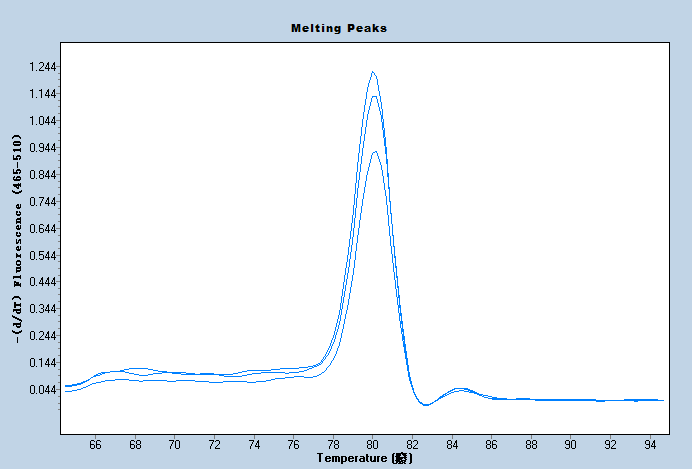


***Lc.10.2141***


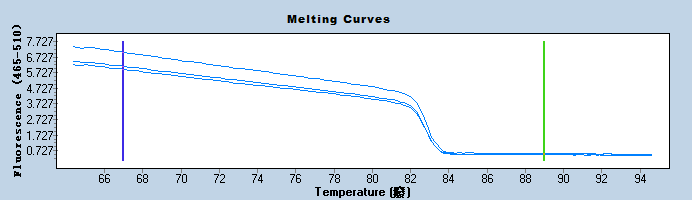

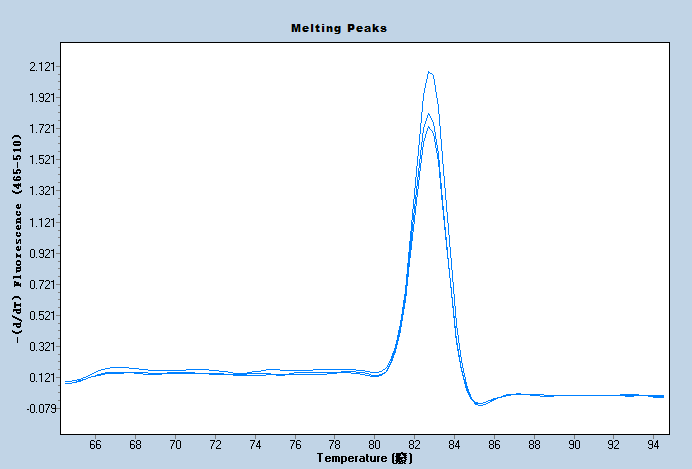


***Lc.11.1628***

**
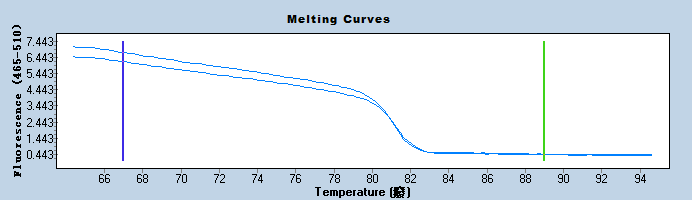
*
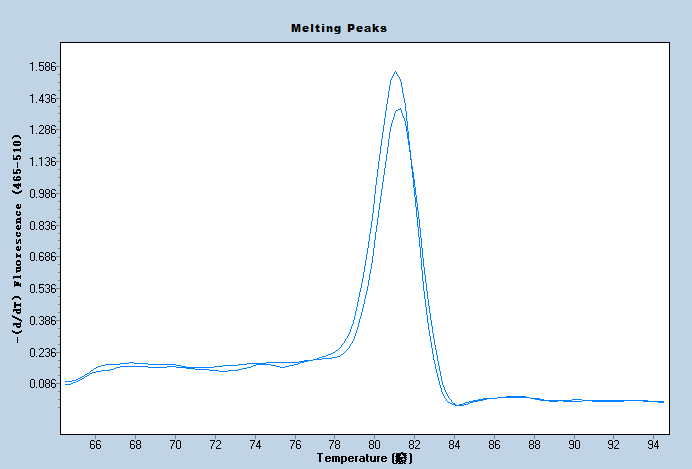
***

***Lc.11.1938***

***Lc.12.1389***


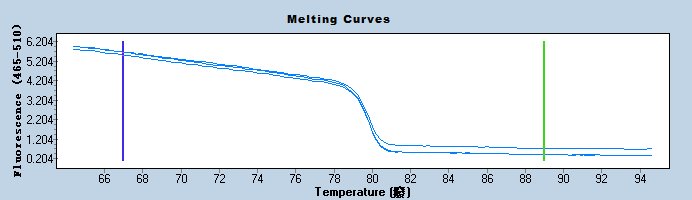

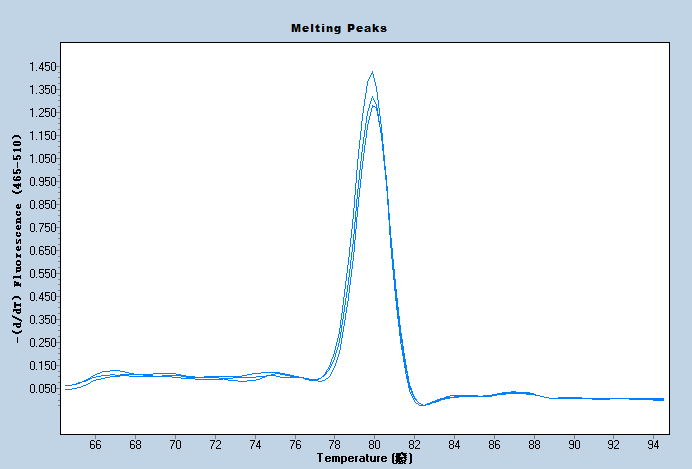


***Lc.13.1106***


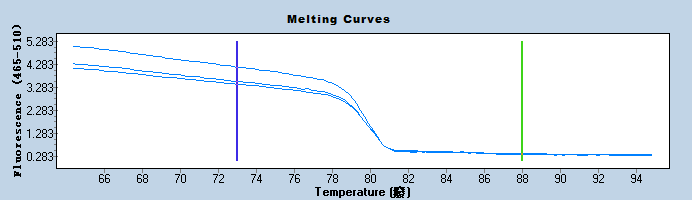

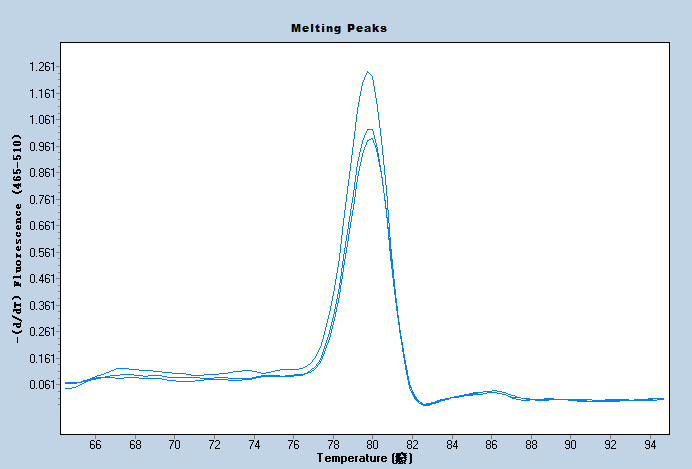


***Lc.13.1548***


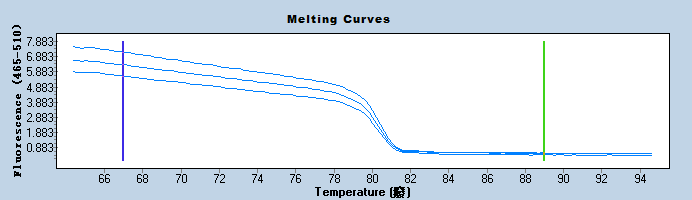

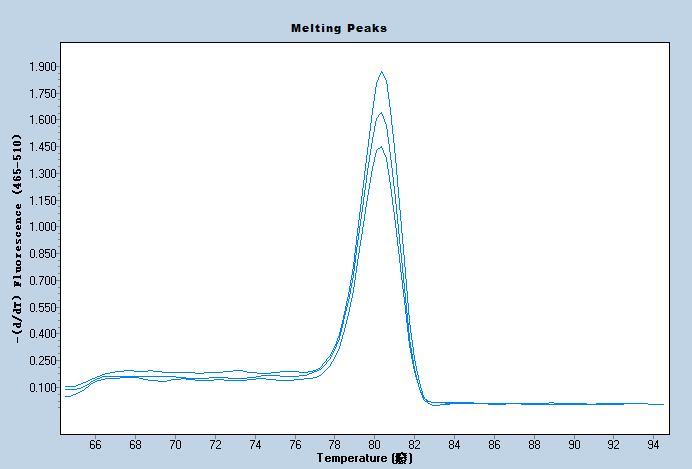


***Lc.13.1561***


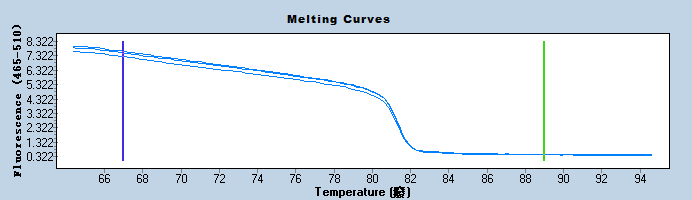

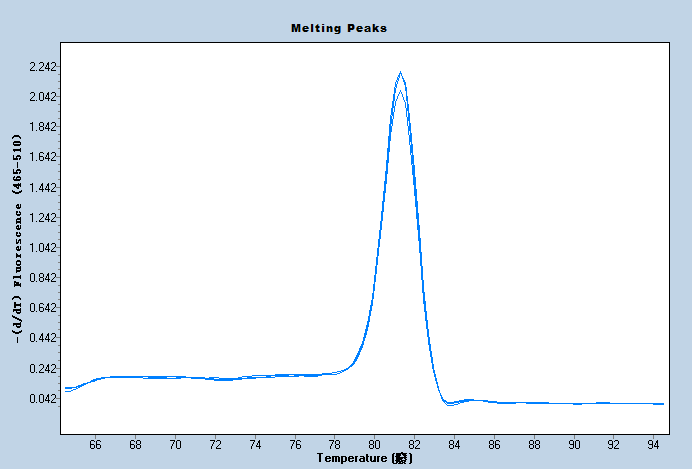


***Lc.14.1455***

**
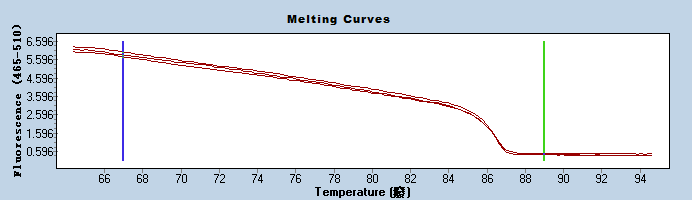
*
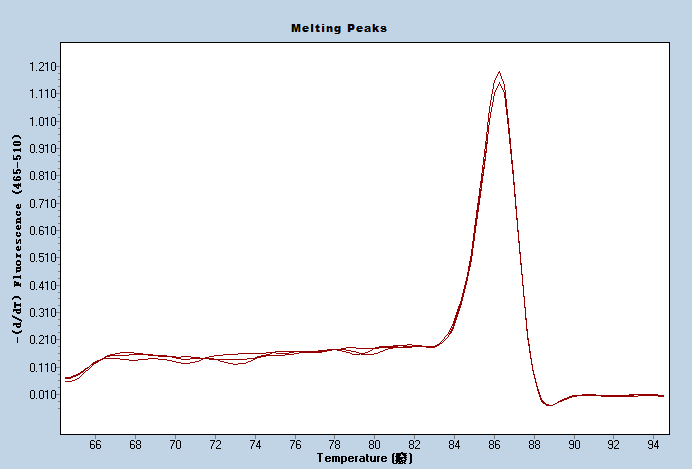
***

***Lc.14.1926***

**
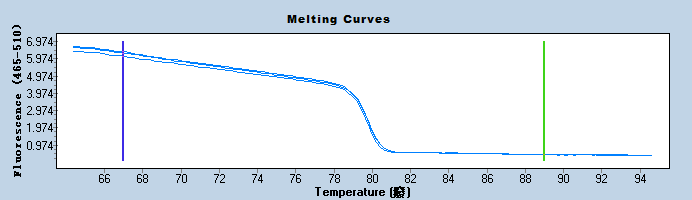
*
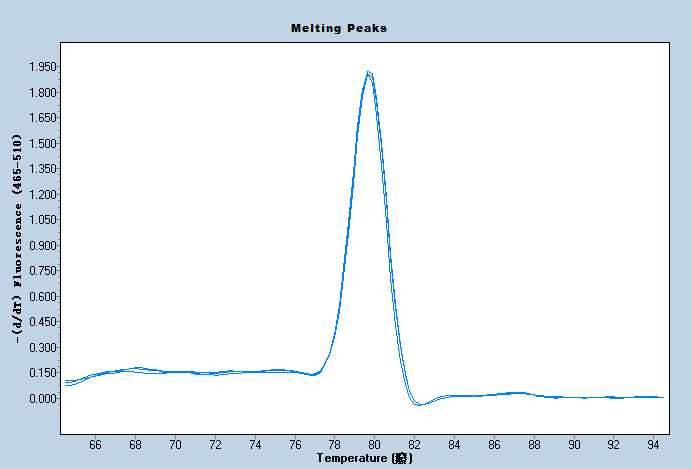
***

***Lc.13.1454***


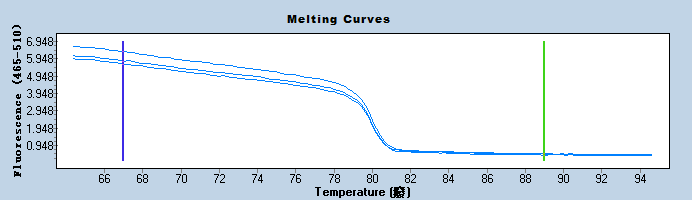

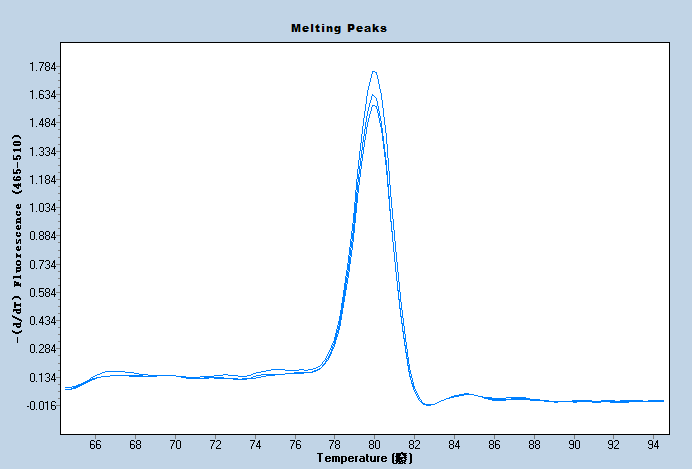


***Lc.1.511***

**
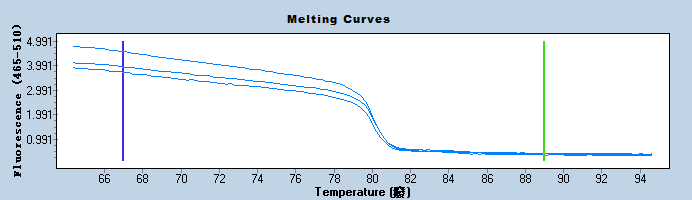
** ***
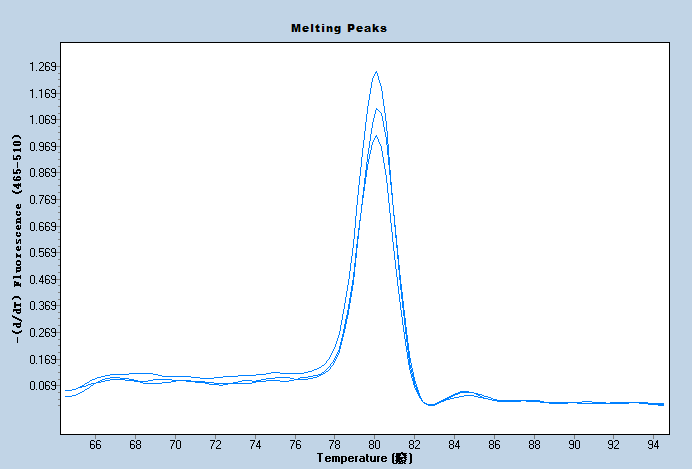
***

***Lc.2.3184***


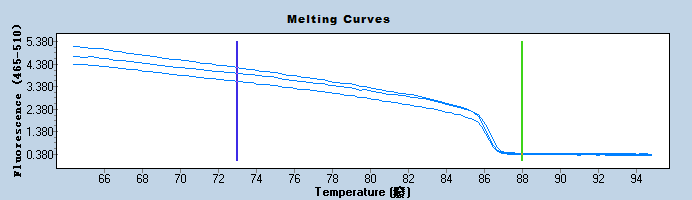

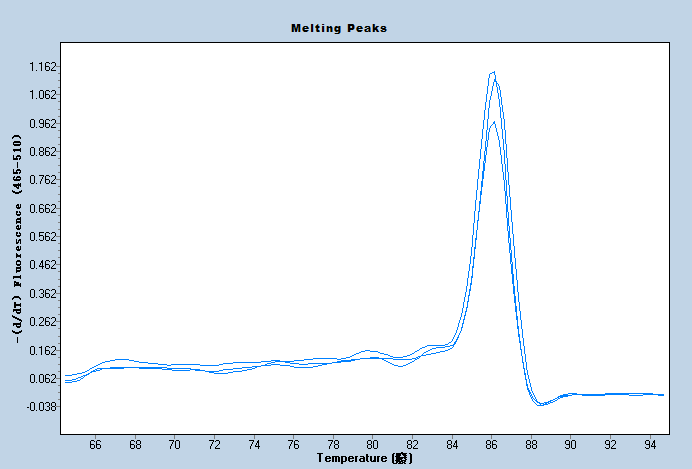


***Lc.2.424***


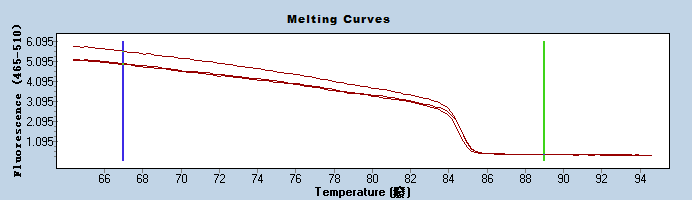

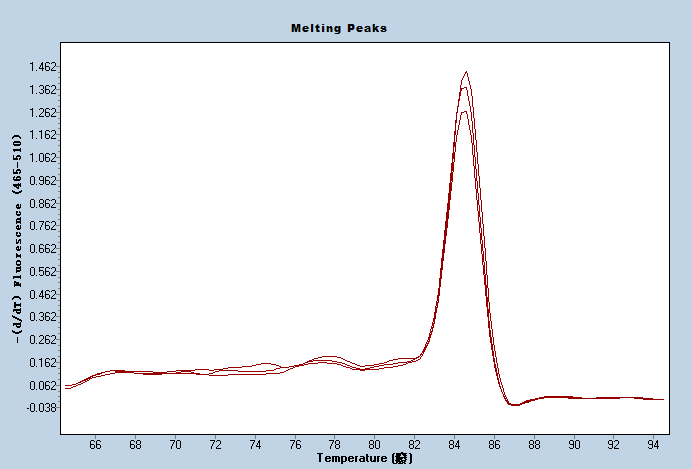


***Lc2.994***

**
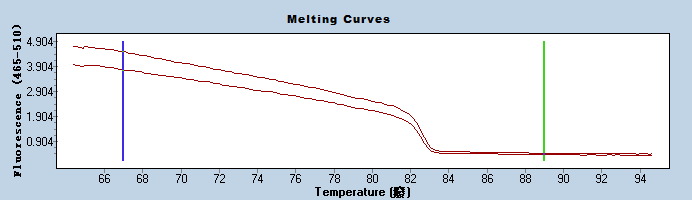
** ***
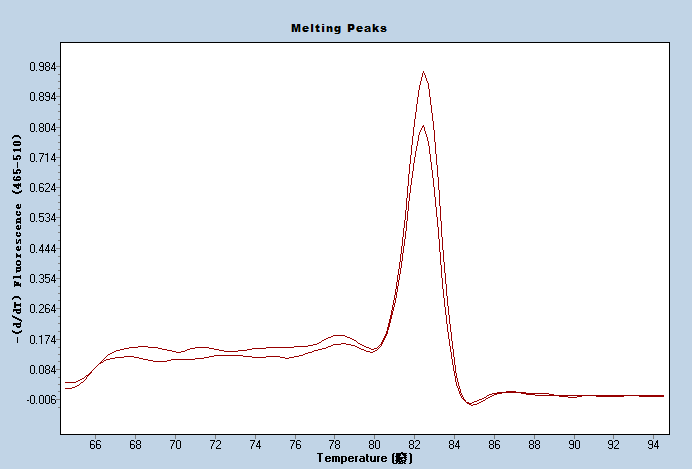
***

***Lc.4.1474***


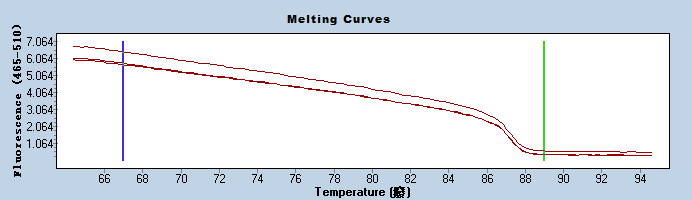

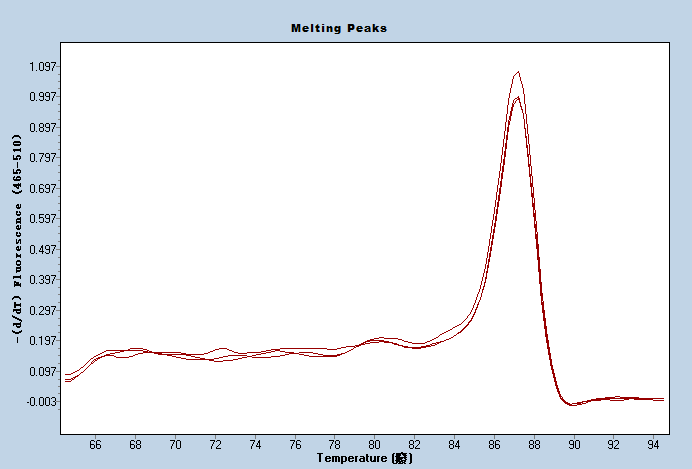


***Lc.4.1918***


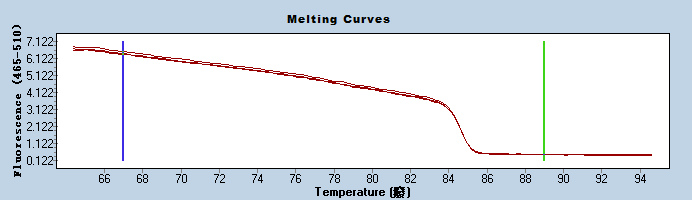

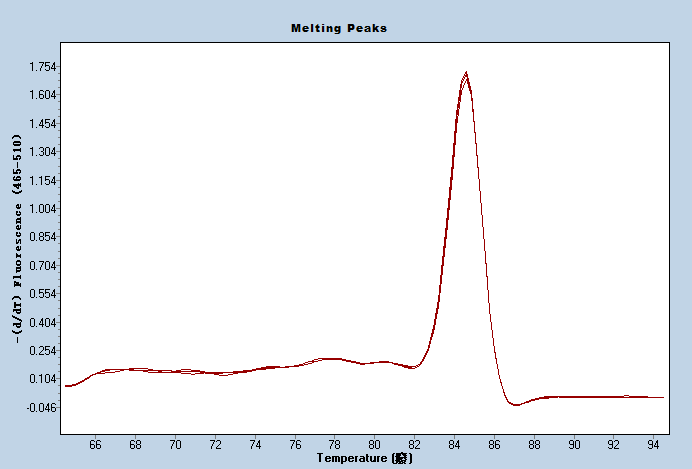


***Lc.7.818***


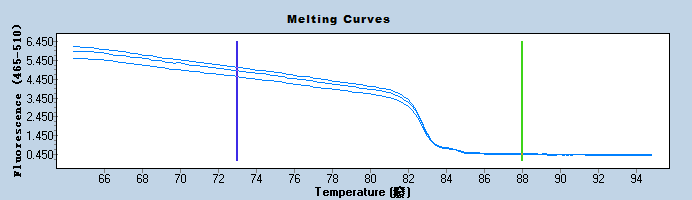

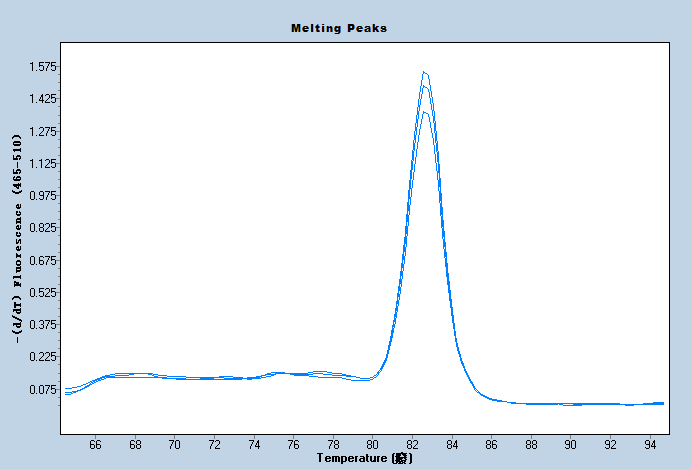


***Lc.8.1213***


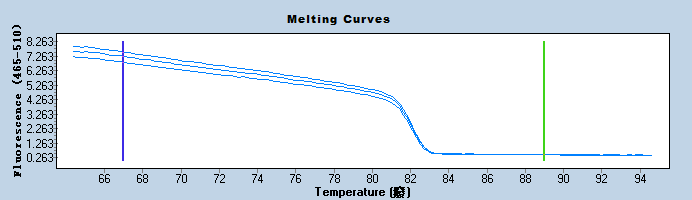

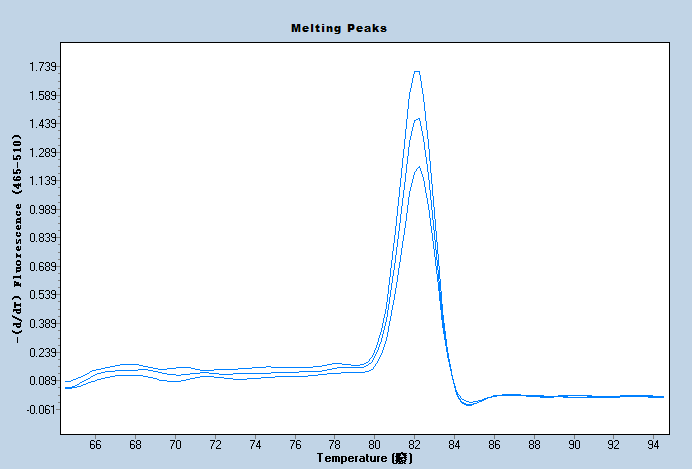


***Lc.9.1824***


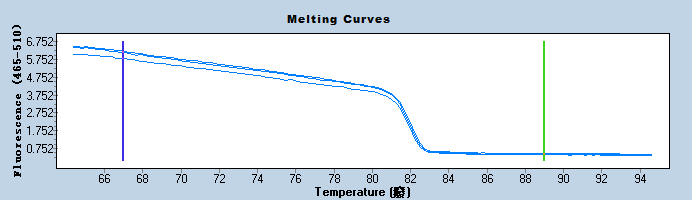

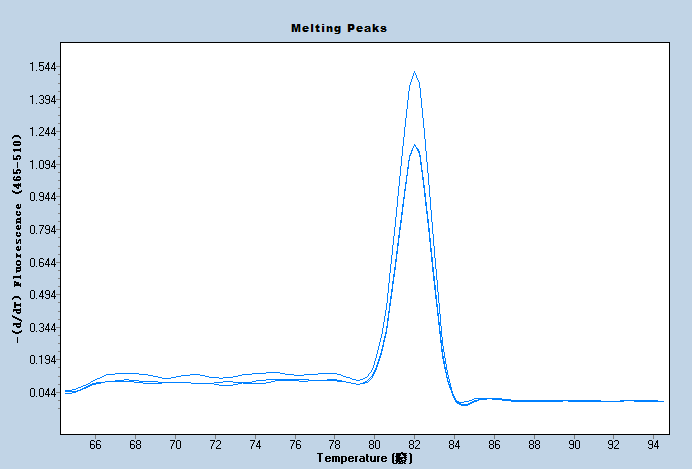


***LcEF1α(elongation factor 1-alpha )***


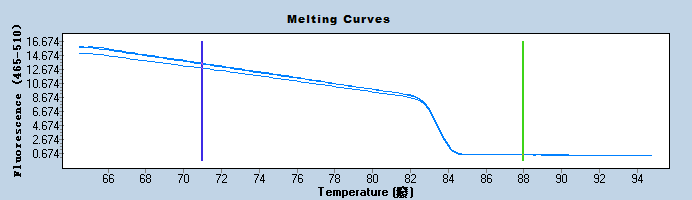

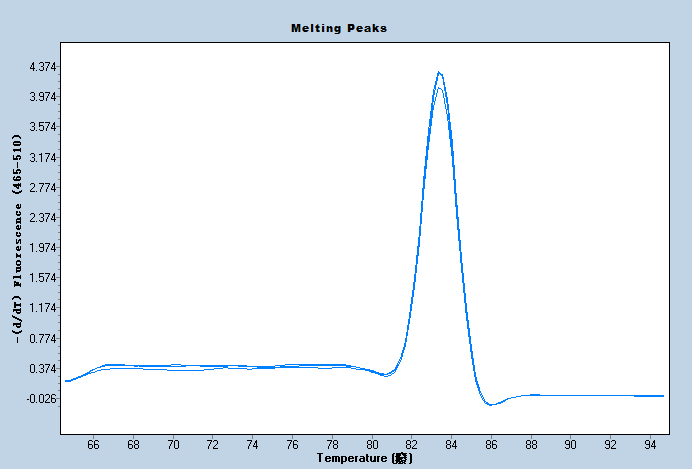


***LcActin***

**
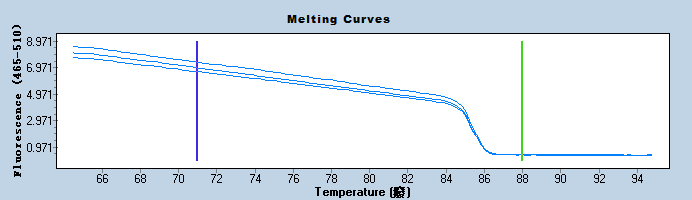
*
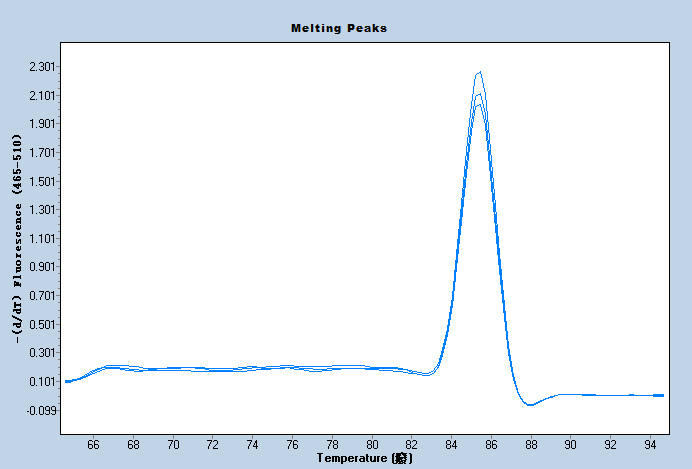
***
